# Tooth crown tissue proportions and enamel thickness in Early Pleistocene *Homo antecessor* molars (Atapuerca, Spain)

**DOI:** 10.1371/journal.pone.0203334

**Published:** 2018-10-03

**Authors:** Laura Martín-Francés, María Martinón-Torres, Marina Martínez de Pinillos, Cecilia García-Campos, Mario Modesto-Mata, Clément Zanolli, Laura Rodríguez, José María Bermúdez de Castro

**Affiliations:** 1 Univ. Bordeaux, CNRS, MCC, PACEA, UMR 5199 F_33615, Pessac, France; 2 Centro Nacional de Investigación sobre la Evolución Humana, Burgos, Spain; 3 Anthropology Department, University College London, London, United Kingdom; 4 Laboratoire AMIS, UMR 5288 CNRS, Université Toulouse III Paul Sabatier, Toulouse, France; 5 Laboratorio de Evolución Humana, Departamento de Ciencias Históricas y Geografía, Universidad de Burgos, Edificio I+D+i, Burgos, Spain; 6 Facultad de Ciencias de la Salud. Universidad Isabel I, Burgos, Spain; University of Florence, ITALY

## Abstract

Tooth crown tissue proportions and enamel thickness distribution are considered reliable characters for inferring taxonomic identity, phylogenetic relationships, dietary and behavioural adaptations in fossil and extant hominids. While most Pleistocene hominins display variations from thick to hyper-thick enamel, Neanderthals exhibit relatively thinner. However, the chronological and geographical origin for the appearance of this typical Neanderthal condition is still unknown. The European late Early Pleistocene species *Homo antecessor* (Gran Dolina-TD6 site, Sierra de Atapuerca) represents an opportunity to investigate the appearance of the thin condition in the fossil record. In this study, we aim to test the hypothesis if *H*. *antecessor* molars approximates the Neanderthal condition for tissue proportions and enamel thickness. To do so, for the first time we characterised the molar inner structural organization in this Early Pleistocene hominin taxon (n = 17) and compared it to extinct and extant populations of the genus *Homo* from African, Asian and European origin (n = 355). The comparative sample includes maxillary and mandibular molars belonging to *H*. *erectus*, East and North African *Homo*, European Middle Pleistocene *Homo*, Neanderthals, and fossil and extant *H*. *sapiens*. We used high-resolution images to investigate the endostructural configuration of TD6 molars (tissue proportions, enamel thickness and distribution). TD6 permanent molars tend to exhibit on average thick absolute and relative enamel in 2D and 3D estimates, both in the complete crown and the lateral enamel. This condition is shared with the majority of extinct and extant hominin sample, except for Neanderthals and some isolated specimens. However, while the total crown percentage of dentine in TD6 globally resembles the low modern values, the lateral crown percentage of dentine tends to be much higher, closer to the Neanderthal signal. Similarly, the *H*. *antecessor* molar enamel distribution maps reveal a relative distribution pattern that is more similar to the Neanderthal condition (with the thickest enamel more spread at the periphery of the occlusal basin) rather than that of other fossil specimens and modern humans (with thicker cuspal enamel). Future studies on European Middle Pleistocene populations will provide more insights into the evolutionary trajectory of the typical Neanderthal dental structural organization.

## Introduction

Tooth crown tissue proportions and enamel thickness distribution are considered reliable characters for inferring taxonomic identity, phylogenetic relationships, dietary and behavioural adaptations in fossil and extant hominids [1–16]. Advances in virtual paleoanthropology evinced the existence of a temporal trend in the internal tooth structural organization within the European Neanderthal lineage [3, 8, 10, 13, 14, 17–26]. In particular, the relative thin enamel condition documented in Neanderthals is likely linked to odontogenetic mechanisms, such as a faster developmental trajectory and a more complex topography and larger surface of the EDJ [8, 25, 27, 28]. On the contrary, studies on modern humans related their relative thick enamel to a unique odontogenetic process and extreme dental reduction, likely leading to an allometric trend of increasing enamel thickness [5, 10, 29, 30]. Still the geographical and chronological context for the appearance of the thin condition is unknown. In this way, the characterization of the structural organization of dental remains belonging to European species predating Neanderthals is of great importance for understanding the variability of this trait.

*Homo antecessor* assemblage was recovered from the Gran Dolina-TD6 cave site of Sierra de Atapuerca (northern Spain) and dated *ca*. 0.8–0.9 Ma [31–35]. Recent studies [36–38] support previous hypothesis that placed TD6 hominins close to the node of divergence of both Neanderthals and modern humans [39], or some time after the split [40, 41]. *H*. *antecessor* distinctiveness derives from its mosaicism of characters [39]. Together with cranial and postcranial synapomorphies [38, 42–45], *H*. *antecessor* dentition displays dental derived features shared with Eurasian and African Middle Pleistocene *Homo*, Neanderthals and modern humans. Moreover, these studies showed higher resemblance between *H*. *antecessor* and Neanderthals in the upper dentition, opposed to the apparently more primitive conformation of the *H*. *antecessor* lower dentition [38, 46–51]. Therefore, TD6 hypodigm represents an excellent opportunity to investigate if a relatively thinner enamel condition is already detectable in this European Early Pleistocene assemblage. The objective of this study is to test the hypothesis if *H*. *antecessor* molars approximates the Neanderthal rather than the *H*. *sapiens* condition for tissue proportions and enamel thickness. To do so, for the first time we will provide the molar tissue estimates in this Early Pleistocene species through the application of standard virtual anthropology protocols.

## Materials and methods

In total, this study includes 372 molars belonging to extinct and extant populations of different chronologies and geographic origin. The systematic excavation of the upper levels of Gran Dolina initiated in 1981. In 1993, the excavation of a 6m^2^ biostratigraphic survey pit started, reaching level TD6 in 1994. This level excavated during four consecutive years, yielded skeletal and dental remains, assigned to *Homo antecessor*, in association with lithic and faunal remains [52]. The Atapuerca Research Team obtained all the permits for the excavation and study of the Atapuerca complex, including Gran Dolina, which complied with all regulations from Dirección General de Patrimonio-Junta de Castilla y León. At present, the complete fossil assemblage is deposited at the Museo de Burgos (Spain). The *H*. *antecessor* molar sample comprises 19 specimens, 11 mandibular and eight maxillary elements representing all molar classes [38]. In this study, we excluded two teeth belonging to individual H3 [53], its upper right M1 because of its more advanced wear degree than the antimere and its upper left M3 due to the too early stage of development (the crown is not fully formed). Therefore, we analysed 17 molars, 11 mandibular and six maxillary, belonging to *H*. *antecessor* (Table 1). Moreover, and for comparative purposes, the study included 355 molars belonging to extinct and extant populations of the genus *Homo* of African, Asian and European origin. Part of the comparative material and associated data (measurements) used for the 2D and 3D (complete molar crown) enamel thickness was extracted from the literature (Table 1). However, data on both 3D complete crown and lateral enamel thickness is limited. Therefore, we also included molars (n = 88; original data) belonging to *H*. *erectus* from Sangiran (n = 4), North African *Homo* from Tighenif (n = 3), East African *Homo* from Eritrea (n = 1), European Middle Pleistocene *Homo* from Fontana Ranuccio and Visogliano (n = 2), Neanderthals (n = 26) and modern humans (n = 52) from different origins (see Table 1). The Neanderthal sample, including the specimens from Regourdou, Krapina, Abri Suard and Abri Bourgeois-Delaunay, is available online in the NESPOS database. Finally, the *H*. *sapiens* molar sample comprises collections of modern humans of European origin deposited at the Muséum national d'Histoire naturelle of Paris (France), University of Toulouse, University of Poitiers, Museo di Storia Naturale di Trieste (Italy), Institut of Anatomy of Strasbourg (France) and University College of London (U.K.) (see Table 1 and S1–S3 Tables).

**Table 1:** Fossil and recent comparative samples used for crown 2D and 3D complete crown and lateral 3D measurements.

**Samples**	**N**	**Tooth**	**Specimens**	**References/Institution**
*H*. *antecessor* (TD6)	4	UM1	Atapuerca-Gran Dolina: ATD6-10, ATD6-11, ATD6-69, ATD6-103	Original data/Museo de Burgos (Spain)
*H*. *erectus* (HER)	1		Sangiran: NG91-G10nº1	Zanolli [54]
North African *Homo* (NAH)	1		Tighenif: UM1	Zanolli and Mazurier [11]
European Middle Pleistocene *Homo* (EMPH)	2		Steinheim: UM1	Smith et al. [10]
			Visogliano: UM2	Zanolli et al. [17]
Neanderthal (NEA)	11		Engis: UM1	Olejniczak et al.[8]
			El Sidrón: SR1105	
			Le Moustier: UM1	
			Scladina: SCLA_4A_4	
			La Quina: UM1	
			Krapina: KRD134, KRD101, D136, D171, D174, D16	Original data from Nespos
Modern humans (MH)	43		Modern humans from South Africa, North America and Europe (n = 37)	Olejniczak et al.[8] ; Smith et al. [9, 10] and Smith *pers*. *comm*.
			Modern humans from Europe (n = 6):	Original data/ Museo di Storia Naturale di Trieste (Italy)
			Bosco Pontini 6 ULM1	
			Bosco Pontini 6 URM1	
			Bosco Pontini 9	
			Bosco Pontini 8	
			Pigorini ULM1	
			Pigorini ULM1b	
*H*. *antecessor* (TD6)	2	UM2	Atapuerca-Gran Dolina: ATD6-12, ATD6-69	Original data/Museo de Burgos (Spain)
*H*. *erectus* (HER)	2		Apothecary Collection China: CA 771	Smith et al. [10]
			Hexian: PA833	Xing et al. [50]
North African *Homo* (NAH)	1		Thomas Quarry	Smith et al. [10]
European Middle Pleistocene *Homo* (EMPH)	2		Steinheim: UM2	Smith et al. [10]
			Visogliano3: UM2	Zanolli et al. [17]
Neanderthal (NEA)	14		El Sidrón: SR332, SR4, SR531, SR551	Olejniczak et al.[8]
			Le Moustier: UM2	
			Scladina: SCLA_4A_3	
			Spy: Spy I	Bayle et al. [55]
			La Quina: UM2	
			Krapina: KRD98, D96, D135, D165, D166, D169	Original data from Nespos website
Fossil *H*. *sapiens* (FHS)	1		Qafzeh: UM2	Smith et al. [9, 10]
Modern humans (MH)	30		Modern humans from South Africa, North America and Europe (n = 25)	Olejniczak et al.[8] ; Smith et al. [9, 10] and Smith *pers*. *comm*.
			Modern humans from Europe (n = 5):	Original data/ Museo Nazionale Preistorico Etnografico Luigi Pigorini of Rome (Italy)
			Bosco Pontini 5	
			Bosco Pontini 6 URM2	
			Bosco Pontini 9	
			Pigorini URM2	
			Pigorini ULM2	
**Samples**	**N**	**Tooth**	**Specimens**	**References/Institution**
*H*. *antecessor* (TD6)	4	LM1	Atapuerca-Gran Dolina: ATD6-5, ATD6-94, ATD6-112, ATD6-96	Original data/Museo de Burgos (Spain)
East African *Homo* (EAH)	1		Buia: MA 93	Zanolli et al. [56]
North African *Homo* (NAH)	1		Tighenif: LM1	Zanolli and Mazurier [11]
*H*. *erectus* (HER)	1		Sangiran: NG92.2	Zanolli [54]
European Middle Pleistocene *Homo* (EMPH)	1		Fontana Ranuccio: FR1R	Zanolli et al. [17]
Neanderthal (NEA)	22		Roc de Marsal: LM1 (2)	Olejniczak et al. [8]
			Engis: LM1	
			Ehringsdorf: G-1048-69	
			El Sidrón: SR755, SR540 Le Moustier: LM1	
			Regoudou: LM1	
			Scladina: SCLA_4A_1	
			Abri Suard: S5, S49, S14-7	
			Abri Bourgeois-Delaunay: BDJ4C9	
			Combe Grenal: CG IV	
			Krapina: KRP53, KRP54, KRP55, KRPD80, D77, D79, D81, D105	Original data from Nespos website
Modern humans (MH)	72		Modern humans from South Africa, North America and Europe (n = 55)	Olejniczak et al.[8]; Smith et al. [9, 10] and Smith *pers*. *comm*.
			Modern humans from Europe (n = 17):	Original data/ Museo di Storia Naturale di Trieste (Italy)
			Bosco Pontini 1	
			Bosco Pontini 2	
			San Canziano	
			San Canziano	
			Val Rosandra	
			B996-scht1	Original data/ Muséum nationald'Histoire Naturelle of Paris (France)
			B996-scht2	
			B998-scht1-mand2	
			B998-scht2-mand3	
			B998-scht2-mand1	
			MH-UdP	Original data/ University of Poitiers (France)
			MH-UdP	
			UTP	
			U21	Institute of Anatomy of Strasbourg, (France)
			U57	
			Sbg2	
			Sbg4	
*H*. *antecessor* (TD6)	3	LM2	Atapuerca-Gran Dolina: ATD6-5, ATD6-144, ATD6-96	Original data/Museo de Burgos (Spain)
North African *Homo* (NAH)	1		Tighenif: Tighenif_2	Zanolli and Mazurier [11]
*H*. *erectus* (HER)	4		Sangiran: NG0802.3, NG92.3, NG92D6ZE57s/d76, NG0802.2	Zanolli [54]
Neanderthal (NEA)	13		Abri Suard: S36	Olejniczak et al.[8]
			Krapina: KRD6, KRD10 (2), KRP55	Original data from Nespos website
			KRP54, D86, D107, D1	
			Le Moustier: LM2	
			Regourdou: LM2 (2)	
			Scladina: SCLA_4A_1	
Modern humans (MH)	62		Modern humans from South Africa, North America and Europe (n = 46)	Olejniczak et al. [8]; Smith et al. [9, 10]; Smith *pers*. *comm*.; Weber and Bookstein [57]
			Modern humans from Europe and Africa (n = 16):	Original data/ Museo di Storia Naturale di Trieste (Italy)
			Bosco Pontini 1	
			Bosco Pontini 2	
			Bosco Pontini 4	
			San Canziano	
			San Canziano	
			Val Rosandra	
			MH-UdP	Original data/ University of Poitiers (France)
			MH-UTP	
			B996-24852	Original data/ Muséum national d'Histoire Naturelle of Paris (France)
			B996-scht1	
			B996-scht1	
			B998-scht1mand2	
			B995	
			MH-MNHN	
			UCL 108	Original data/ University College of London (UK)
			UCL 107	
*H*. *antecessor* (TD6)	3	LM3	Atapuerca-Gran Dolina: ATD6-5, ATD6-113, ATD6-96	Original data/Museo de Burgos (Spain)
North African *Homo* (NAH)	1		Tighenif: Tighenif_2	Zanolli and Mazurier [11]
*H*. *erectus* (HER)	1		Sangiran: NG9107.2	Zanolli [54]
European Middle Pleistocene *Homo* (EMPH)	1		Mauer: LM3	Smith et al. [10]
			Mala Balanica: BH-1	Skinner et al. [24]
Neanderthal (NEA)	15		Abri Suard: S36, S43	Olejniczak et al. [8]
			Krapina: KRD9, KRP57, KRPD85, KRD5, KRD7, KDR106	Original data from Nespos
			Le Moustier: LM3 (2)	
			Regourdou: LM3 (2)	
			La Quina: Q760-H9	
			Abri Bourgeois-Delaunay: BD01	
			Combe Grenal: CG XII	
Modern humans (MH)	52		Modern humans from South Africa, North America and Europe (n = 44)	Olejniczak et al. [8] ; Smith et al. [9, 10] and Smith *pers*. *comm*.
			Modern humans from Europe (n = 8):	Original data/ Muséum national d'Histoire Naturelle of Paris (France)
			B998-scht3	
			B996-scht1	
			B996-scht1	
			B996-scht2 M3g	
			B998-scht2-mand2	
			MH-CZ	Original data/ University of Toulouse (France)
			MH-UdP	Original data/ University of Poitiers (France)
			San Canziano	Original data/ Museo di Storia Naturale di Trieste (Italy)

Microtomographic scanning (mCT) of the samples was performed at the CENIEH facilities. Isolated fossil teeth were scanned with Scanco Medical Micro-CT80 system, using the following parameters: 70 kV and 114 mA, 0.1 Al filter and resulting isometric voxel size 18μm. In addition, the molars included in mandibular and/or maxillary fragments were mCT scanned with a GE 103 Phoenix v/tome/x_s 240 instrument with a different set of parameters ranging from 120-140kV and 140μA, 0.2 Cu filter and resulting isometric voxel size ranging from 27 to 36μm.

We assessed the wear degree following Molnar’s categories [58]. All molars exhibited wear scores between 1 (no wear) and 3 (small dentine patches). Due to the lack of a reproducible protocol to reconstruct enamel cusps and dentine horns in 3D, we excluded molars exhibiting wear 3 from the total crown volume analyses, but we included them in our investigation of the lateral tissue proportions.

We performed the virtual sectioning of the molars following the protocol described in Olejniczak and colleagues [8]. The mCT image stack was imported into Amira (6.3.0, FEI Inc.) and rotated into anatomical position. Then, the tip of three dentine horns (protoconid, metaconid and hypoconid in the mandibular molars and protocone, paracone and metacone in the maxillary molars) were identified and the image stack was adjusted to intersect these three points of interest. Finally, a new plane perpendicular to the plane containing the three dentine horns was rotated to pass through the mesial dentine horns (protoconid and metaconid in the mandibular molars and protocone and paracone in the maxillary molars). We assessed enamel thickness from virtual 2D mesial cross-section planes in each TD6 molar as described in [1, 59] using Amira (6.2, FEI Inc.) and ImageJ (1.51, NIH). In each mesial plane, we measured the enamel (c) and dentine cap (b, including the pulp) areas (in mm^2^), adding up into the total crown area (a, in mm^2^), and the enamel-dentine junction (EDJ) length (d, in mm). We calculated the average enamel thickness (AET = c/d), the relative enamel thickness (RET = 100*AET/(b^1/2^)) and the percentage of dentine and pulp in the molar crown (b/a = 100*b/a in %). We performed enamel cusp reconstruction in 11 molars following Smith et al. [60] protocol. We selected the cross-section plane of an unworn TD6 molar exhibiting the closest morphology to the worn molar. Following, we superimposed the contour of the unworn molar on top of the worn molar and draw the contour using Photoshop CC 2018 (S1 Fig). We used ImageJ (1.51, NIH) software to take the measurements in the reconstructed molar cap. Inter- and intra-observer error was assessed by LMF and MMP. They performed the complete process, including orientation of the specimen, mesial plane definition, measures of the variables in each TD6 specimen. Each set of measurements was repeated in three alternative days, inter- and intra-observer error resulted in < 4%.

We assessed volume enamel thickness of the molar caps in 13 specimens, excluding ATD6-94, ATD6-96, ATD6-10 and ATD6-11 due to their more advanced occlusal wear showing dentine exposure (wear degree 3 following [58]). Using Amira (6.3.0, FEI Inc.) we performed the segmentation of the dental tissues (enamel, dentine and pulp). We used the semiautomatic tool, threshold-based segmentation, and manual corrections. We employed Olejniczak et al. [8] protocol for the definition of the cervical plane. That is, the plane halfway between the most apical continuous ring of enamel and the plane containing the last hint of enamel. The following variables were measured and/or calculated: volume of the enamel (Ve in mm^3^); volume of the coronal dentine including the pulp enclosed in the crown (Vcdp in mm^3^); total volume of the crown, including the enamel, dentine and pulp (Vc in mm^3^); surface of the EDJ (SEDJ in mm^2^); percentage of dentine and pulp in the total crown volume (Vcdp/Vc = 100*Vcdp/Vc in %); 3D average enamel thickness (3D AET = Ve/SEDJ in mm) and, 3D relative enamel thickness (3D RET = 100*3D AET/(Vcdp^1/3^) a scale-free measurement) [2, 8].

In order to extract the largest amount of information of the TD6 specimens, including the occlusal worn molars, we assessed lateral (non-occlusal) enamel thickness in the complete sample (n = 17). In Amira (6.3.0, FEI Inc.) we defined the occlusal basin plane, a plane parallel to the cervical plane and tangent to the lowest enamel point of the occlusal basin. Following all material above the occlusal basin plane were removed and only the enamel, dentine and pulp between these two planes were measured [61–63]. The following variables were measured and/or calculated: lateral volume of the enamel (LVe in mm^3^); lateral volume of the coronal dentine including the pulp enclosed in the crown (LVcdp in mm^3^); total lateral volume of the crown, including the lateral enamel, dentine and pulp (LVc in mm^3^); lateral surface of the EDJ (LSEDJ in mm^2^); percentage of dentine and pulp in the lateral crown volume (LVcdp/LVc = 100*LVcdp/LVc in %); 3D average enamel thickness (3D LAET = LVe/LSEDJ in mm) and, 3D lateral relative enamel thickness (3D LRET = 100*3D LAET/(LVcdp^1/3^) a scale-free measurement) [63].

The results of the 2D and 3D measurements in TD6 specimens were compared with two populations, Neanderthals and modern humans (MH). Adjusted Z-scores [64, 65] of the three variables accounting for tissue proportions (percentage of dentine, [3D] AET and [3D] RET) were computed to compare 2D and 3D dental tissue proportions and enamel thickness values of the TD6 specimens to the means and standard deviations of the Neanderthal and MH groups. This statistical method allows the comparison of unbalanced samples by using Student’s inverse t distribution. In these Z-scores the -1.0 to +1.0 interval comprises the 95% of the variation in the reference sample. In addition, standard box and whisker plots were computed to represent three set of variables of crown volume and lateral volume (including 3D Vcdp/Vc, 3D AET, 3D RET and 3D LVcdp/Vc, 3D LAET, 3D LRET) in the TD6 sample and the complete comparative specimens and/or groups.

In order to visualize enamel thickness topographic distribution in TD6, 3D chromatic maps were generated in Amira (6.3.0, FEI Inc.). The defined chromatic scale is from thinnest (blue) to thickest (red) [20, 26]. For comparative purposes, we generated the chromatic maps of a selected sample of specimens of European and African origin, including: Neanderthal from La Chaise de Vouthon (lower M1 and M3) and Krapina (lower M1 and upper M1 and M2); modern humans (all molar classes); Tighenif (lower M2 and M3) and Eritrea (lower M1).

## Ethics statement

This study concerns the analysis of an original fossil human dental sample constituted by seventeen isolated specimens. The specimens are stored at the Museo de Burgos Spain. José María Bermúdez de Castro, co-director of the Atapuerca Research Team, and María Martinón Torres, director of the CENIEH and research member of the Atapuerca Research Team, have made possible this study within the framework of a long-term scientific project in the field of paleoanthropology. In addition, the modern human collection comprises European samples are deposited in several European institutions, including the Muséum national d'Histoire naturelle of Paris (France), University of Toulouse, University of Poitiers, Museo di Storia Naturale di Trieste (Italy), Institute of Anatomy of Strasbourg (France) and University College of London (U.K.). Access to these collections are granted through scientific collaborations and all necessary permits were obtained for the described study, which complied with all relevant regulations.

## Results

### Tooth tissue proportions and enamel thickness

**Fig 1:**
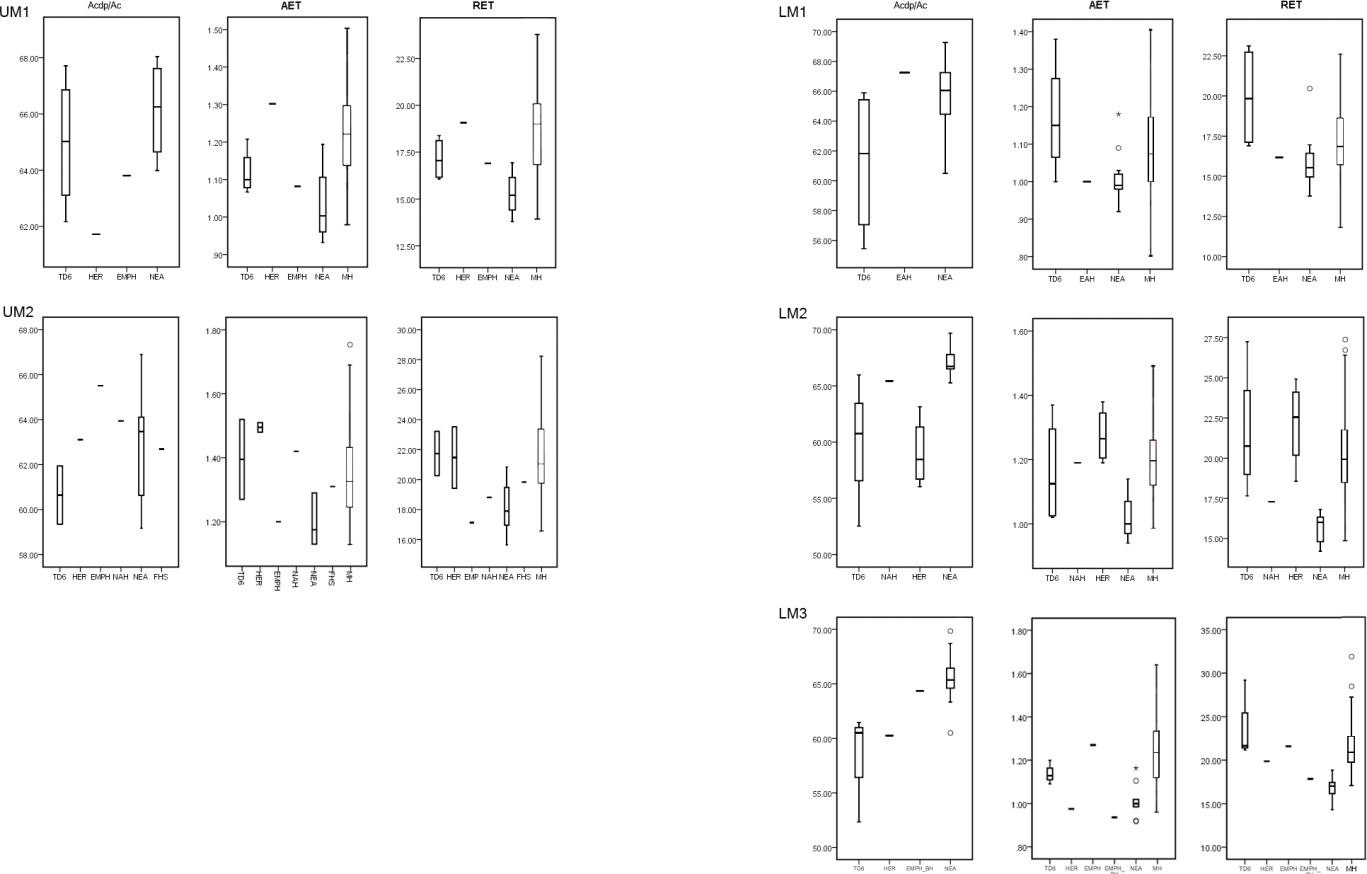
Box Plots depicting 2D values. Values of the percentage of dentine and pulp (b/a), average enamel thickness (AET) and relative enamel thickness (RET) in the maxillary (left) and mandibular (right) molars of the TD6 and the comparative samples. (Modern human boxplots modified from Smith et al., [10]).

**Fig 2:**
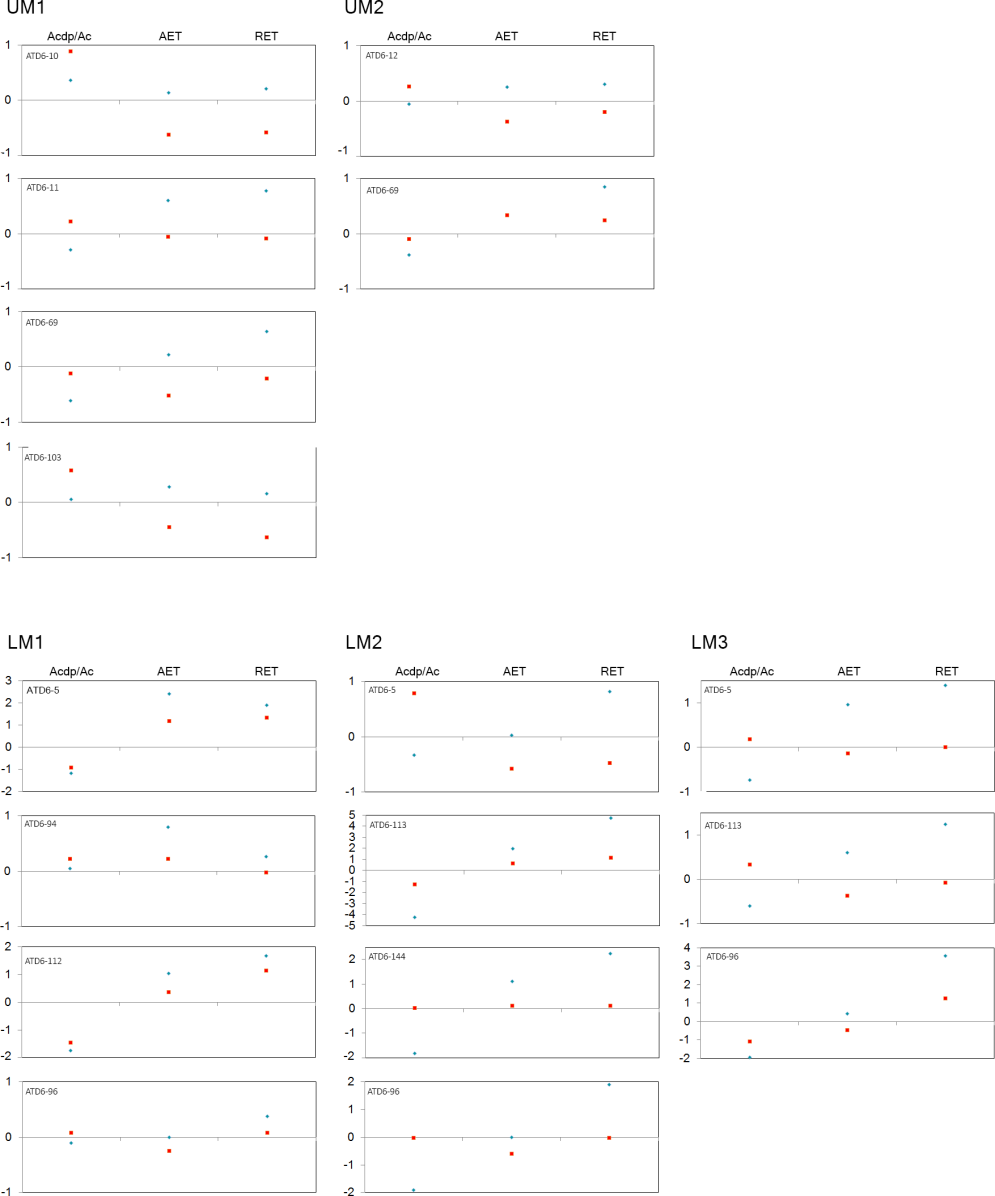
Adjusted Z-scores of 2D variables. Z-scores graphs for the percentage of dentine and pulp (b/a), average enamel thickness (AET) and relative enamel thickness (RET) in the TD6 maxillary (top) and mandibular (bottom) molars and compared to the variation expressed by Neanderthals (blue diamonds) and modern humans (red squares). The solid line passing through zero represents the mean, and the -1 and 1 values correspond to the 95% limit of variation expressed for the two comparative samples.

**Fig 3:**
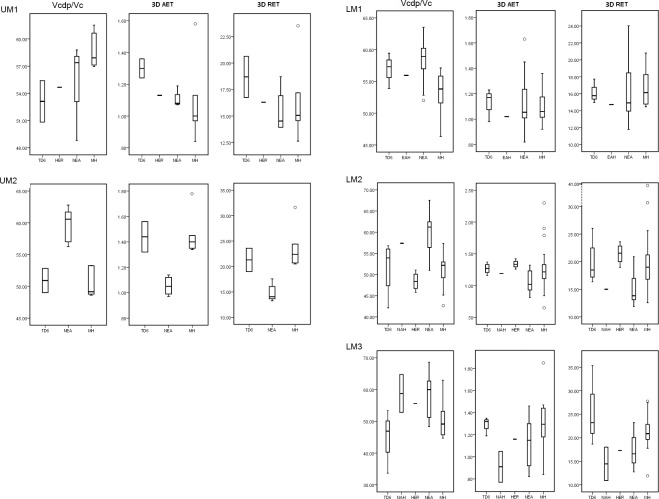
Box plots of the 3D, complete crown, values. 3D values depicting the percentage of dentine and pulp in the total crown volume (Vcdp/Vc), average enamel thickness (3D AET) and relative enamel thickness (3D RET) in maxillary (left) and mandibular (right) molars of the TD6 and the comparative specimens/samples.

**Fig 4:**
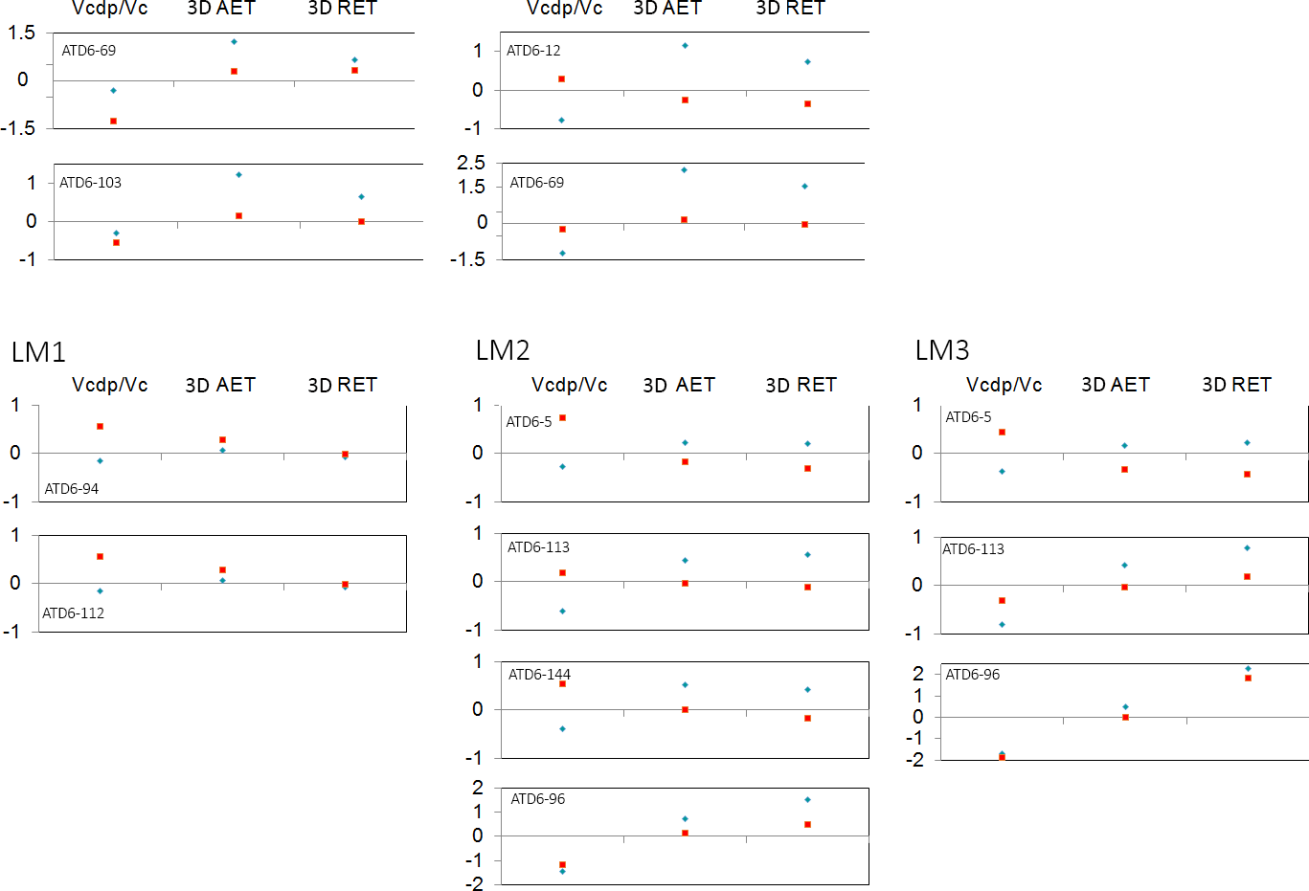
Adjusted Z-scores of 3D, complete crown, variables. Z-score graphs for the crown percentage of dentine and pulp (Vcdp/Vc), average enamel thickness (3D AET) and relative enamel thickness (3D RET) in the TD6 maxillary (top) and mandibular (bottom) molars and compared to the variation expressed by Neanderthals (blue diamonds) and modern humans (red squares). The solid line passing through zero represents the mean, and the -1 and 1 values correspond to the 95% limit of variation expressed for the two comparative samples.

Tissue proportions assessed for the TD6 maxillary and mandibular molars and the comparative samples are shown in Tables 2–4 and Figs 1–4 (for the complete set of values of the entire sample, including TD6 and comparative material see S1–S3 Tables).

**Table 2:** 2D enamel thickness variables assessed in TD6 maxillary and mandibular molars and compared with extinct and extant specimens/populations. Individual values and mean and range (in bold) are given for TD6 molar data. Mean and range are given for the comparative sample when having more than two specimens. Individual values are given for the rest of comparative sample.

**Specimens**	**Tooth**	**N**	**Mean & Range**	**c (mm**^**2**^**)**	**b (mm**^**2**^**)**	**e (mm)**	**b/a (%)**	**AET (mm)**	**RET**
	UM1								
ATD6-10		1		20.52	43.04	19.24	67.72	1.07	16.26
ATD6-11		1		24.23	43.17	20.06	64.05	1.21	18.38
ATD6-69		1		22.72	37.33	20.84	62.16	1.09	17.84
ATD6-103		1		24.56	47.66	22.14	65.99	1.11	16.07
			**Mean**	**23.01**	**42.80**	**20.57**	**64.98**	**1.12**	**17.14**
			**Range**	**20.52–24.56**	**37.33–47.66**	**19.24–22.14**	**62.16–67.72**	**1.07–1.21**	**16.07–18.38**
HER		1		28.90	46.60	22.20	61.72	1.30	19.07
EMPH		1		23.25	40.99	21.49	63.81	1.08	16.90
NEA		5	Mean	22.98	43.94	22.36	65.68	1.03	15.50
			Range	21.03–28.0	37.19–49.75	21.08–23.45	63.87–68.03	0.93–1.19	13.79–16.92
MH		37	Mean	42.87	25.18	20.64	62.85	1.22	18.75
			Range	32.46–59.44		17.66–24.11	57.16–68.98	0.98–1.50	13.95–23.86
	UM2								
ATD6-12		1		23.99	39.05	18.95	61.94	1.27	20.26
ATD6-69		1		29.33	42.83	19.30	59.35	1.52	23.22
			**Mean**	**26.66**	**40.94**	**19.13**	**60.65**	**1.39**	**21.74**
			**Range**	**23.99–29.33**	**39.05–42.83**	**18.95–19.30**	**59.35–61.94**	**1.27–1.52**	**20.26–23.22**
HER		1		33.73	57.70	22.86	63.11	1.48	19.42
HER		1						1.51	23.52
EMPH		1		25.79	48.99	21.51	65.51	1.20	17.13
NAH		1		32.02	56.78	22.59	63.94	1.42	18.81
NEA		7	Mean	25.96	43.41	21.40	62.36	1.21	18.59
			Range	22.72–29.05	36.74–58.72	19.85–24.22	58.77–66.90	1.12–1.29	15.65–21.41
FHS		1		25.97	43.63	19.82	62.69	1.31	19.84
MH		25	Mean	42.76	28.61	20.49	60.05	1.40	21.59
			Range	30.12–65.71		18.22–24.83	53.80–66.45	1.13–1.76	16.49–28.03
**Specimens**	**Tooth**	**N**	**Mean & Range**	**c (mm**^**2**^**)**	**b (mm**^**2**^**)**	**e (mm)**	**b/a (%)**	**AET (mm)**	**RET**
	LM1								
ATD6-5		1		25.02	35.52	18.16	58.67	1.38	23.12
ATD6-94		1		23.12	44.67	20.47	65.89	1.13	16.90
ATD6-112		1		21.98	27.35	18.82	55.44	1.17	22.33
ATD6-96		1		18.07	33.52	18.00	64.97	1.00	17.34
			**Mean**	**22.05**	**35.27**	**18.86**	**61.25**	**1.17**	**19.92**
			**Range**	**18.07–25.02**	**27.35–44.67**	**18.00–20.47**	**55.44–65.89**	**1.00–1.38**	**16.90–23.12**
EAH		1		18.60	38.20	18.60	67.25	1.00	16.18
NEA		12	Mean	21.12	40.44	21.07	65.62	1.00	15.88
			Range	16.82–23.81	33.38–45.42	17.65–23.25	60.48–69.27	0.92–1.18	13.76–20.45
MH		55	Mean	40.16	21.74	20.32	64.48	1.07	16.99
			Range	27.45–50.82		16.73–22.94	59.19–72.65	0.80–1.40	11.76–22.62
	LM2								
ATD6-5		1		17.40	33.74	16.97	65.98	1.03	17.65
ATD6-113		1		21.32	33.22	17.47	60.91	1.22	21.17
ATD6-144		1		22.98	25.43	16.72	52.53	1.37	27.25
ATD6-96		1		16.34	25.14	16.03	60.61	1.02	20.33
			**Mean**	**19.51**	**29.38**	**16.80**	**60.01**	**1.16**	**21.60**
			**Range**	**16.34–22.98**	**25.14–33.74**	**16.03–16.97**	**52.53–65.98**	**1.002–1.37**	**17.65–27.25**
NAH		1		25.20	47.70	21.10	65.43	1.19	17.29
HER		4	Mean	23.18	33.65	18.23	59.03	1.27	22.15
			Range	21.2–23.4	30.60–41.30	17.40–20.20	56.04–59.54	1.19–1.37	18.56–24.93
NEA		8	Mean	20.97	42.73	20.49	67.13	1.02	15.66
			Range	17.1–22.53	33.85–47.56	17.78–22.89	65.27–67.95	0.94–1.14	14.2016.80
MH		45	Mean	34.33	22.05	18.52	60.78	1.19	20.51
			Range	23.75–42.24		15.22–21.60	53.21–67.80	0.94–1.55	14.85–27.66
	LM3								
ATD6-5		1		20.00	30.65	16.68	60.51	1.20	21.66
ATD6-113		1		17.83	28.45	15.80	61.47	1.13	21.16
ATD6-96		1		12.72	13.97	11.66	52.34	1.09	29.19
			**Mean**	**16.85**	**24.36**	**14.71**	**58.11**	**1.14**	**24.00**
			**Range**	**12.72–20.00**	**13.97–30.65**	**11.66–16.68**	**52.33–61.47**	**1.09–1.20**	**29.19–21.16**
HER		1		15.90	24.10	16.30	60.25	0.98	19.87
EMPH		1						1.27	21.60
EMPH_BH		1		15.22	27.48	16.26	64.36	0.94	17.86
NEA		9	Mean	19.28	36.71	18.99	65.45	1.01	16.80
			Range	16.70–24.84	30.58–48.02	17.56–20.93	63.34–69.83	0.91–1.16	14.29–18.86
MH		44	Mean	33.09	22.58	18.27	59.31	1.24	21.63
			Range	24.40–45.98	16.75–29.42	15.90–22.26	50.82–64.61	0.98–1.67	17.22–31.84

Upper molars. TD6: *H*. *antecessor* from Gran Dolina (original data). HER: *H*. *erectus* (Sangiran_M1, Zanolli [54]; China_M2, Smith et al. [10]; Xing et al. [67]). EMPH: European Middle Pleistocene *Homo* (Steinheim_M1, Smith et al. [10]). NAH: North African *Homo* (Thomas Quarry_M2, Smith et al. [10]). NEA: Neanderthals (Olejniczak et al. [8]). FHS: fossil *H*. *sapiens* (Qafzeh_M2, Smith et al. [10]). MH: modern humans (Smith et al. [9, 10] and *pers*. *comm*.). Lower molars TD6: *H*. *antecessor* from Gran Dolina (original data). EAH: East African *Homo* (Eritrea_M1, Zanolli et al. [56]). NAH: North African *Homo* (Tighenif_M2, Zanolli and Mazurier [11]). HER: *H*. *erectus* (Sangiran_M2 & M3; Zanolli [54]). EMPH: European Middle Pleistocene *Homo* (Mauer_M3, Smith et al. [10]. EMPH_BH: European Middle Pleistocene *Homo* (Mala Balanica_M3, Skinner et al. [24]). NEA: Neanderthals (Olejniczak et al. [8]). MH: modern humans (Smith et al. [9, 10] and Smith *pers*. *comm*.).

**Table 3:** 3D enamel thickness variables assessed in TD6 maxillary and mandibular molars and compared with extinct and extant specimens/populations. Individual values and mean and range (in bold) are given for TD6 molar data. Mean and range are given for the comparative sample when having more than two specimens. Individual values are given for the rest of comparative sample.

**Specimens**	**Tooth**	**N**		**Ve (mm**^**3**^**)**	**Vcdp (mm**^**3**^**)**	**SEDJ (mm**^**2**^**)**	**Vcdp/Vc (%)**	**3D AET (mm)**	**3D RET**
	UM1								
AT6-69		1		279.20	288.59	204.69	50.83	1.36	20.64
AT6-103		1		324.29	402.75	262.22	55.40	1.24	16.75
			**Mean**	**301.75**	**345.67**	**233.45**	**53.11**	**1.30**	**18.69**
			**Range**	**279.20–324.29**	**288.402.75**	**204.69–262.22**	**50.83–55.40**	**1.24–1.36**	**16.75–20.64**
HER		1		275.00	331.60	243.80	54.67	1.13	16.30
NEA		4	Mean	298.19	387.32	271.40	55.58	1.11	15.43
			Range	249.44–341.92	272.93–460.43	217.26–317.81	48.79–58.77	1.07–1.19	13.93–18.71
MH		5	Mean	206.89	294.65	195.14	58.83	1.10	16.59
			Range	185.21–229.89	285.79–304.79	145.36–255.00	56.96–61.52	0.84–1.58	12.63–23.52
	UM2								
AT6-12		1		298.30	334.00	226.17	52.82	1.32	19.01
AT6-69		1		301.65	289.98	193.17	49.01	1.56	23.59
			**Mean**	**299.98**	**311.99**	**209.67**	**50.92**	**1.44**	**21.30**
			**Range**	**298.30–301.65**	**289.98–334.00**	**193.17–226.17**	**49.01–52.82**	**1.32–1.56**	**19.01–23.59**
NEA		5	Mean	240.33	359.34	229.64	59.67	1.05	14.91
			Range	210.06–256.45	270.23–418.51	185.00–250.31	56.26–62.80	0.96–1.13	13.24–17.56
MH		5	Mean	231.66	238.18	161.66	50.62	1.46	23.93
			Range	188.55–293.86	179.58–284.08	105.67–218.18	48.60–53.60	1.34–1.78	20.49–31.63
**Specimens**	**Tooth**	**N**		**Ve (mm**^**3**^**)**	**Vcdp (mm**^**3**^**)**	**SEDJ (mm**^**2**^**)**	**Vcdp/Vc (%)**	**3D AET (mm)**	**3D RET**
	LM1								
AT6-94		1		303.96	408.04	259.92	57.31	1.17	15.77
AT6-112		1		282.92	330.75	230.67	53.90	1.23	17.74
			**Mean**	**293.44**	**369.40**	**245.29**	**55.60**	**1.20**	**16.75**
			**Range**	**282.92–303.96**	**330.75–386.27**	**230.67–259.92**	**53.90–57.31**	**1.17–1.23**	**15.77–17.74**
EAH		1		261.40	332.20	256.10	55.96	1.02	14.74
NEA		12	Mean	245.48	346.93	223.31	223.31	1.13	16.29
			Range	189.76–309.52	246.35–447.75	129.33–302.55	129.33–302.55	0.81–1.63	11.79–24.01
MH		12	Mean	247.31	286.78	227.11	227.11	1.09	16.67
			Range	199.59–349.88	172.59–420.84	172.29–271.13	172.29–271.13	0.92–1.36	14.46–2081
	LM2								
AT6-5		1		273.25	359.31	234.89	56.80	1.16	16.36
AT6-113		1		259.81	288.47	207.70	52.61	1.25	18.93
AT6-144		1		292.18	360.80	227.24	55.25	1.29	18.06
ATD6-96		1		200.92	146.35	146.44	42.14	1.37	26.04
			**Mean**	**256.54**	**288.73**	**204.07**	**51.70**	**1.27**	**19.85**
			**Range**	**200.92–292.81**	**146.35–360.80**	**146.44–234.89**	**42.14–56.80**	**1.16–1.37**	**16.36–26.04**
NAH		1		373.06	502.42	312.54	57.39	1.19	15.01
HER		4	Mean	261.80	246.95	196.30	48.39	1.34	21.42
			Range	240.8–279.7	219.2–291.9	181.1–222.2	45.76–51.06	1.25–1.42	18.97–23.60
NEA		9	Mean	247.38	373.05	233.40	59.98	1.06	14.93
			Range	169.13–308.44	250.03–519.34	182.48–302.91	50.96–67.53	0.81–1.31	11.88–20.92
MH		26	Mean	248.70	262.97	203.25	51.11	1.27	20.11
			Range	161.94–361.91	135.4–426.01	95.4–285.35	42.65–57.32	0.65–2.3	12.56–40.71
	LM3								
AT6-5		1		229.21	261.93	191.97	53.33	1.19	18.66
ATD6-96		1		109.39	55.52	81.15	33.67	1.35	35.33
AT6-113		1		210.24	185.64	158.77	46.89	1.32	23.21
			**Mean**	**182.95**	**167.70**	**143.96**	**44.63**	**1.29**	**25.74**
			**Range**	**109.39–229.21**	**55.52–261.93**	**81.15–191.97**	**33.67–53.33**	**1.19–1.35**	**18.66–35.33**
NAH		1		372.24	371.53	255.48	49.95	1.46	20.27
NEA		9	Mean	208.03	301.62	188.37	58.88	1.11	16.75
			Range	154.04–262.18	244.62–485.90	142.85–228.03	49.11–68.62	0.82–1.40	12.73–22.28
MH		8	Mean	243.62	244.58	182.72	49.49	1.34	21.85
			Range	166.58–362.14	146.81–405.48	121.09–273.48	44.70–55.44	1.08–1.85	17.78–27.77

Upper molars: TD6: *H*. *antecessor* from Gran Dolina (original data). HER: *H*. *erectus* (Sangiran_M1, Zanolli [54]). NEA: Neanderthals (Olejniczak et al. [8]; Bayle et al. [55]. MH: modern humans (Olejniczak et al. [8]). Lower molars: TD6: *H*. *antecessor* from Gran Dolina (original data). EAH: East African *Homo* (Eritrea_M1, Zanolli et al. [56]). NAH: North African *Homo* (Tighenif_M2&M3, Zanolli and Mazurier [11]). HER: *H*. *erectus* (Sangiran, Zanolli [54]). NEA: Neanderthals (Olejniczak et al. [8]). MH: modern humans (Olejniczak et al.[8]; Weber and Bookstein [57] and original data).

**Table 4:** 3D lateral enamel thickness variables assessed in TD6 maxillary and mandibular molars and compared with extinct and extant specimens/populations. Individual values and mean and range (in bold) are given for TD6 molar data. Mean and range are given for the comparative sample when having more than two specimens. Individual values are given for the rest of comparative sample.

**Specimens**	**Tooth**	**N**		**LVe (mm**^**3**^**)**	**LVcdp (mm**^**3**^**)**	**LSEDJ (mm**^**2**^**)**	**LVcdp/Vc (%)**	**3D LAET (mm)**	**3D LRET**
	UM1								
ATD6-10		1		79.59	270.66	110.49	77.28	0.72	11.14
ATD6-11		1		83.46	305.42	123.32	78.54	0.68	10.05
ATD6-69		1		71.81	235.23	99.59	76.61	0.72	11.68
ATD6-103		1		71.91	324.14	126.00	81.84	0.57	8.31
			**Mean**	**76.69**	**283.86**	**114.85**	**78.57**	**0.67**	**10.29**
			**Range**	**71.81–83.46**	**235.23–324.14**	**99.59–126.00**	**76.61–81.84**	**0.57–0.72**	**8.31–11.68**
NAH		1		116.99	398.79	172.25	77.32	0.68	9.23
EMPH		1		86.20	264.45	121.41	75.42	0.71	11.06
NEA		6	Mean	87.69	301.66	132.08	77.49	0.66	9.86
			Range	51.29–112.46	210.21–414.76	96.00–167.39	74.82–80.39	0.53–0.72	8.99–10.93
MH		6	Mean	65.99	208.11	113.54	75.95	0.58	9.79
			Range	56.40–74.20	186.41–225.37	104.52–118.59	74.77–78.06	0.54–0.63	8.91–10.28
ATD6-12		1	UM2	66.08	274.03	105.42	80.57	0.63	9.65
ATD6-69		1		61.77	224.72	84.60	78.44	0.73	12.01
			**Mean**	**63.93**	**249.38**	**95.01**	**79.51**	**0.68**	**10.83**
			**Range**	**61.77–66.08**	**224.72–274.03**	**84.60–405.42**	**78.44–80.57**	**0.63–0.73**	**9.65–12.01**
EMPH		1		76.34	242.16	134.09	76.03	0.57	9.13
NEA		6	Mean	72.88	330.35	115.88	79.94	0.62	9.40
			Range	56.04–94.32	199.03–678.03	100.46–140.79	74.60–91.43	0.56–0.70	6.63–11.03
MH		5	Mean	35.39	132.52	72.26	79.17	0.48	9.45
			Range	24.50–53.52	95.62–171.71	60.46–92.65	76.24–81.00	0.41–0.58	8.86–10.39
**Specimens**	**Tooth**	**N**		**LVe (mm3)**	**LVcdp (mm3)**	**LSEDJ (mm2)**	**LVcdp/Vc (%)**	**3D LAET (mm)**	**3D LRET**
	LM1								
AT6-5		1		59.71	250.86	100.013	80.77	0.60	9.47
AT6-94		1		64.471	333.627	125.71	83.81	0.51	7.39
AT6-112		1		56.72	263.13	106.11	82.27	0.53	8.34
ATD6-96		1		37.48	144.82	58.12	79.44	0.64	12.28
			**Mean**	**54.60**	**248.11**	**97.49**	**81.57**	**0.57**	**9.37**
			**Range**	**37.48–64.47**	**144.82–333.62**	**58.12–125.71**	**79.44–82.27**	**0.51–0.64**	**7.39–12.28**
NAH		1		102.82	389.11	151.14	79.10	0.68	9.32
HER		1		44.20	186.33	85.56	80.83	0.52	9.05
EMPH		1		60.77	239.61	110.08	79.77	0.55	8.89
NEA		10	Mean	74.45	294.03	126.56	80.15	0.58	8.67
			Range	36.07–108.44	205.37–404.52	92.80–159.88	73.84–85.06	0.39–0.79	6.59–11.65
MH		13	Mean	54.71	199.34	95.14	78.54	0.56	9.73
			Range	14.91–113.81	14.91–362.55	38.17–156.12	50.00–82.66	0.39–0.73	7.54–16.60
	LM2								
AT6-5		1		55.69	297.87	103.67	84.25	0.54	8.04
ATD6-96		1		22.26	88.12	35.98	79.83	0.62	13.90
ATD6-144		1		58.74	270.53	107.78	82.16	0.54	8.43
ATD6-113		1		48.47	219.90	83.65	81.94	0.58	9.60
			**Mean**	**46.29**	**219.11**	**82.77**	**82.05**	**0.57**	**9.99**
			**Range**	**22.26–58.74**	**88.12–297.87**	**35.98–107.78**	**79.83–84.25**	**0.54–0.62**	**8.04–13.90**
HER		3	Mean	48.43	210.43	90.71	81.16	0.53	9.03
			Range	42.66–51.89	170.73–244.38	78.85–99.70	80.01–82.48	0.51–0.55	8.48–9.75
NEA		7	Mean	77.99	331.90	130.65	81.28	0.59	8.50
			Range	47.52–110.62	230.45–422.03	93.32–156.14	77.15–84.38	0.43–0.76	6.19–10.11
MH		10	Mean	54.73	205.86	90.49	79.01	0.59	10.19
			Range	32.14–90.83	123.44–351.01	64.89–136.08	74.21–82.60	0.46–0.74	8.45–13.25
	LM3								
ATD6-5		1		39.16	212.36	80.55	84.43	0.49	8.15
ATD6-96		1		6.91	26.48	17.21	79.31	0.40	13.47
ATD6-113		1		25.34	127.31	51.24	83.40	0.49	9.83
			**Mean**	**23.80**	**122.05**	**49.67**	**82.38**	**0.46**	**10.48**
			**Range**	**6.91–39.16**	**26.48–212.36**	**17.21–80.55**	**79.31–84.43**	**0.40–0.49**	**8.15–13.47**
NAH		1		79.89	302.65	124.33	79.12	0.64	9.57
HER		1		29.41	145.13	64.21	83.15	0.46	8.72
NEA		6	Mean	66.21	241.39	111.12	78.44	0.59	9.59
			Range	29.41–83.44	145.13–314.03	64.21–132.28	73.59–83.15	0.46–0.72	8.26–11.65
MH		6	Mean	52.71	210.59	97.40	80.34	0.52	8.82
			Range	22.25–79.90	130.04–314.86	69.99–131.75	77.68–85.39	0.32–0.65	6.28–10.35

Upper molars: TD6: *H*. *antecessor* from Gran Dolina (original data). NAH: North African *Homo* (Tighenif_M2&M3, Zanolli and Mazurier [11]). EMPH: European Middle Pleistocene *Homo* (Visogliano6_M1 & Visogliano3_M2, Zanolli et al. [17]). NEA: Neanderthals (original data). MH: modern humans (European origin, original data). Lower molars: TD6: *H*. *antecessor* from Gran Dolina (original data). NAH: North African *Homo* (Tighenif_M1&M3; Zanolli and Mazurier [11]). HER: *H*. *erectus* (Sangiran_M1&M2; HER: *H*. *erectus* (Sangiran, Zanolli [54]). EMPH: European Middle Pleistocene *Homo* (Fontana Ranuccio_M1, Zanolli et al. [17]). NEA: Neanderthals (original data). MH: modern humans (European origin, original data and Weber and Bookstein [57]).

#### 2D enamel thickness

TD6 maxillary and mandibular molars exhibit rather low 2D values of b/a and EDJ length resulting in thick AET and RET (see Table 2 and Figs 1 and 2). In terms of b/a, for the maxillary M1s, *H*. *antecessor* approximates the condition of late Early to Middle Pleistocene hominin *H*. *erectus* specimens from Sangiran [66] and Steinheim [10], and overlaps with Neanderthals [8, 10] and modern humans (Table 2). For the upper M2s, the TD6 specimens display inferior values than most of the other comparative fossil specimens/samples, even if these overlap with the Neanderthal variation range [8, 10]. Regarding modern humans, the TD6 range encompasses the modern human variability (Table 2). Similar results are found for the lower molars, although wider differences are seen between the *H*. *antecessor* M2s and M3s and the Neanderthals specimens [8]. Similarly, the values of the specimens from North African late Early-early Middle Pleistocene *Homo* specimens of Tighenif [11], as well as the Middle Pleistocene hominin mandibular molars from Mala Balanica [24] are well above the TD6 average value. Conversely, the *H*. *antecessor* mandibular molars values of b/a encompasse the modern human variability (Table 2). For both AET and RET, the *H*. *antecessor* maxillary and mandibular molars exceed or minimally overlap with the superior part of the Neanderthal range of variation [8]. Overall, the TD6 AET and RET values approximate the Javanese *H*. *erectus* condition [54] and overlap or slightly exceed most of the fossil comparative specimens/samples [10, 11, 24, 56, 67]. The modern human range [9, 10] for these two variables encompasses most of the fossil variation, including that of *H*. *antecessor*. The adjusted Z-score analyses (Fig 2) show that the TD6 maxillary specimens are within the 95% of variation of both Neanderthals and modern human groups for the three variables of crown tissue proportion (b/a, AET and RET). For the mandibular molars, eight (ATD6-5 M1, ATD6-112 M1, ATD6-113 M2, ATD6-96 M2, ATD6-144 M2, ATD6-5 M3, ATD6-113 M3, ATD6-96 M3) out of the 11 teeth, lie well beyond the Neanderthal variation for at least one of the three variables. In addition, four mandibular molars (ATD6-5 M1, ATD6-112 M1, ATD6-113 M2, ATD6-96 M3) also exceed the modern human variation for one of these variables.

#### 3D crown enamel thickness

TD6 crown tissue proportions reflect the same pattern described for the 2D results (Table 3 and Figs 3 and 4). The TD6 Vcdp/Vc values for the upper M1 are similar to those of Javanese *H*. *erectus* (Zanolli, 2015). The TD6 values overlap with the Neanderthals at its inferior range ([8] and this study), and are well below the modern human variation [8]. For the upper M2, the *H*. *antecessor* range overlap with that of modern humans. Conversely and due to the low estimates, these groups are quite distant to the Neanderthal estimates [8, 55]. Similar results are found for the lower molars. The TD6 Vcdp/Vc values for the lower molars overlap or exceed in the inferior limit with those of the Neanderthal and modern human variation [8]. Conversely, TD6 Vcdp/Vc values are below the values and range of variation of North African late Early-early *Homo* specimens of Tighenif [11]. The TD6 upper molar 3D AET and 3D RET estimates exceed or slightly overlap with the superior limit of those of the Neanderthal [8] and Javanese *H*. *erectus* [54]. With regard to the modern humans, for the upper M1, 3D AET and 3D RET estimates [8] are lower or slightly overlap with the lower limits of TD6 variation. On the contrary, for the upper M2 modern human estimates [8] overlap or slightly exceeds the superior values of the TD6 variation. The TD6 lower M1 and M2 mean 3D AET and 3D RET values are within the range of the Neanderthal and modern human variation, even if the median value tend to show thicker enamel than Neanderthals and closer to the modern human condition [8]. When compared with other Early-Middle Pleistocene specimens/samples, except for the M2, the *H*. *antecessor* lower molars exceed the values of Javanese *H*. *erectus*, North African *Homo* from Tighenif, and East African *Homo* from Eritrea [11, 54, 56]. The Z-score analysis of maxillary molars (Fig 4) showed that the four maxillary molars lie within the 95% of variation of modern humans for the three variables, except for the percentage of dentine of the upper M1 specimen ATD6-69. On the contrary, only the upper M1 specimen ATD6-103 lies within the Neanderthal variation for the three variables. For the mandibular molars (Fig 4), seven out of nine lie within the 95% of variation of both, modern humans and Neanderthals, and the only two molars outside the range of both groups belong to the same specimen, ATD6-96.

#### 3D lateral enamel thickness

**Fig 5:**
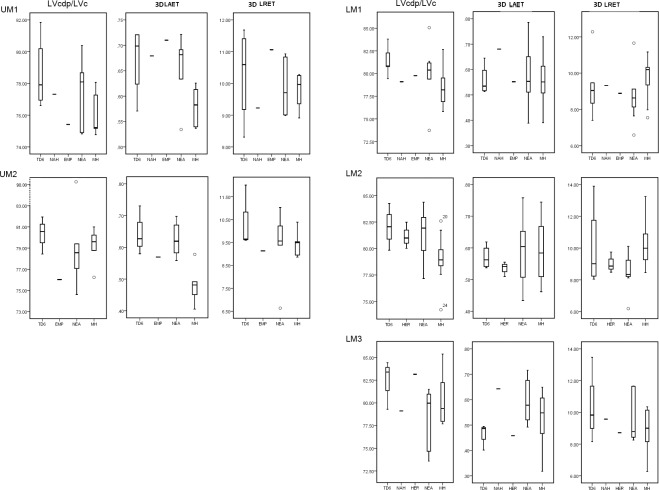
Box plots of the 3D, lateral enamel, values. Box plots depicting lateral percentage of dentine and pulp in the total crown volume (LVcdp/LVc), average enamel thickness (3D LAET) and relative enamel thickness (3D LRET) in maxillary (left) and mandibular (right).

**Fig 6:**
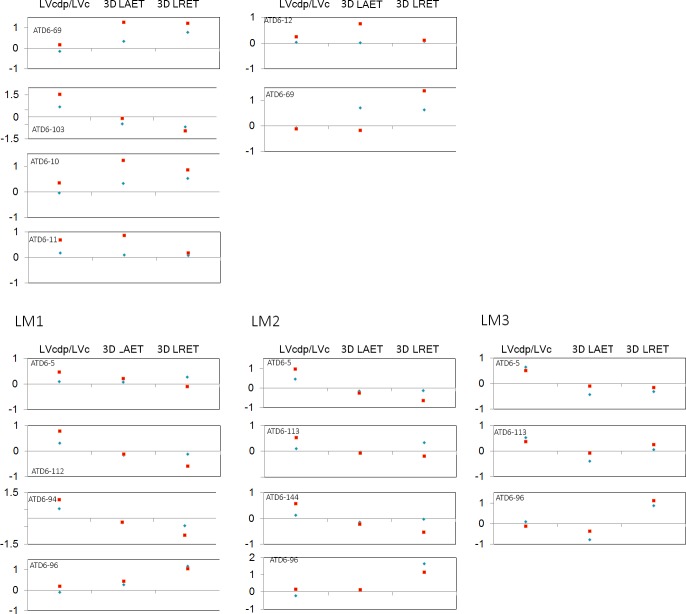
Adjusted Z-scores of 3D, lateral enamel, variables. Z-score graphs for the lateral crown percentage of dentine (right) and pulp in the total crown volume (LVcdp/LVc), average enamel thickness (3D LAET) and relative enamel thickness (3D LRET) in TD6 maxillary (top) and mandibular (bottom) molars and compared to the variation expressed by Neanderthals (blue diamonds) and modern humans (red squares). The solid line passing through zero represents the mean, and the -1 and 1 values correspond to the 95% limit of variation expressed for the two comparative samples.

Results of lateral tissue proportions in TD6 mandibular and maxillary molars revealed a shift in the pattern of tissue proportions compared to what we described for the 2D and 3D crown proportions, although this is more accentuated in the maxillary molars (Table 4 and Figs 5 and 6). The TD6 maxillary molars tend to display higher LVcdp/LVc than the mid-Middle Pleistocene specimens from Visogliano [17], Neanderthals and modern humans, even if overlap with the latter two groups. The value of the late Early-early Middle upper M1 from Tighenif fits within *H*. *antecessor* range. Similar results are found for the lower molars, with the TD6 specimens showing high LVcdp/LVc estimates comparable to Javanese *H*. *erectus*, and overlapping with the Neanderthal and modern human ranges, even though the TD6 average is superior to these two groups. The LVcdp/LVc estimates for Middle Pleistocene isolated lower molars from Tighenif [11] and from Fontana Ranuccio [17] fit with the lower values of the TD6 teeth. Regarding the 3D LAET of the upper molars, *H*. *antecessor* approximates the Neanderthal estimates, with much higher values than those of modern humans. Once scaled through the 3D LRET, the differences between these three groups are less visible as the estimated values overlap. When considering the mandibular molars, the TD6 3D LAET and 3D LRET overlap with all the fossil and extant human groups, except for the 3D LAET of the Middle Pleistocene specimen from Tighenif that exceeds the *H*. *antecessor* range. However, if ATD6-96 molars are not included in the comparison, the mean values of TD6 3D LRET are closer to the mean values of Neanderthals. The Z-score analysis the TD6 specimens (Fig 6) show that the six maxillary molars fall within the 95% of Neanderthal variation for the three variables; on the contrary, three specimens (ATD6-10, ATD6-69, ATD6-103) are outside the 95% of modern human variation. Of the eleven TD6 lower molars analysed, the M1 ATD6-94 is out of the modern human interval, while all the molars of the specimen ATD6-96 show a 3D LRET exceeding both the Neanderthal and modern human ranges.

#### Enamel thickness topographic distribution

**Fig 7:**
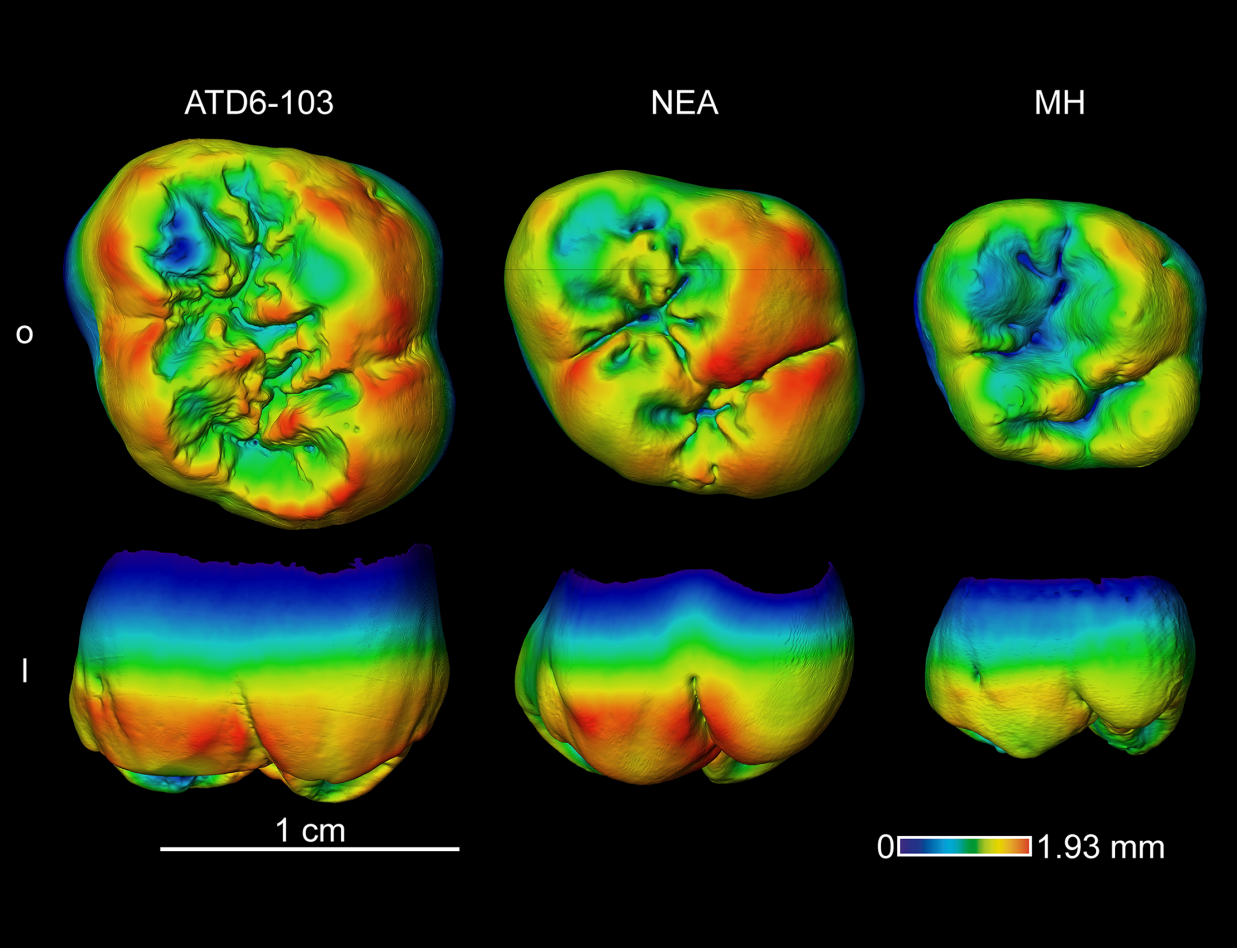
Enamel thickness cartographies of the *H*. *antecessor* upper M1 (ATD6-1103) from Gran Dolina (Atapuerca) compared with those of Neanderthal and modern human. Topographic thickness variation is rendered by a pseudo-colour scale ranging from thinner (dark-blue) to thicker (red). NEA = Neanderthal (Krapina D165) and MH = modern human of European origin (o = occlusal, l = lingual). Scale bar = 1.93 for all specimens.

**Fig 8:**
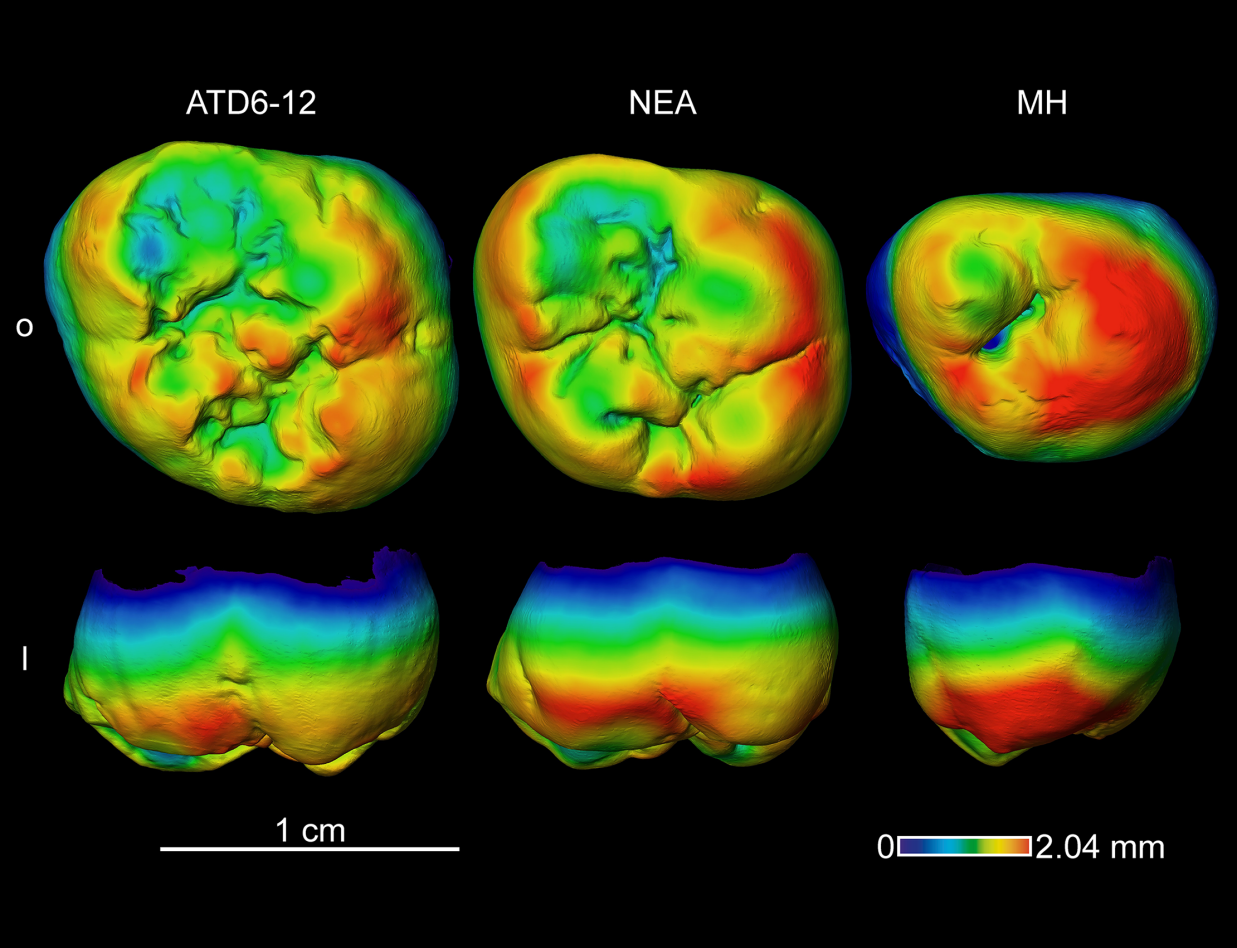
Enamel thickness cartographies of the *H*. *antecessor* upper M2 (ATD6-12) from Gran Dolina (Atapuerca) compared with those of Neanderthal and modern human. Topographic thickness variation is rendered by a pseudo-colour scale ranging from thinner (dark-blue) to thicker (red). NEA = Neanderthal (Krapina D101) and MH = modern human of European origin (o = occlusal, l = lingual). Scale bar = 2.04 for all specimens.

**Fig 9:**
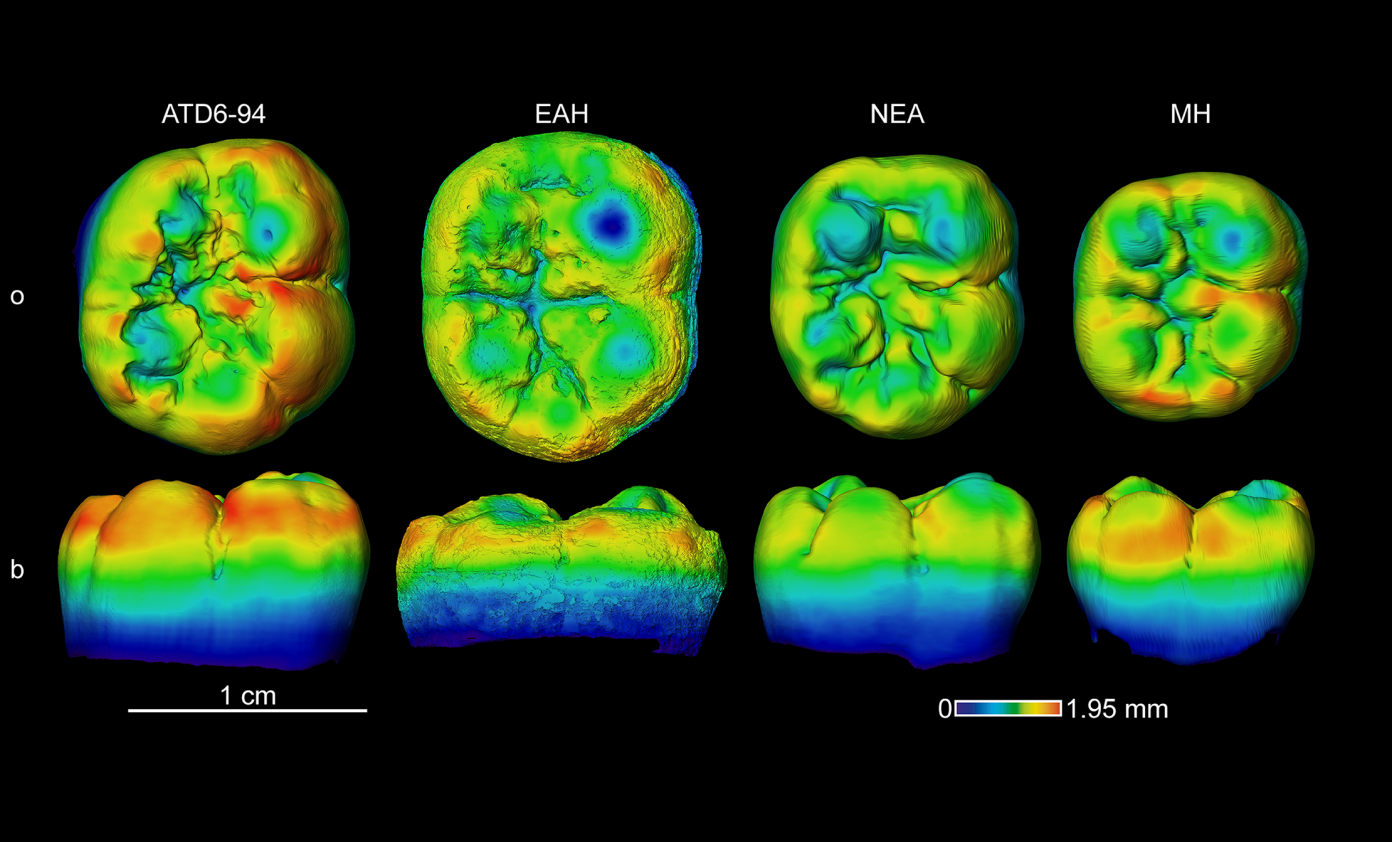
Enamel thickness cartographies of the *H*. *antecessor* lower M1 (ATD6-94) from Gran Dolina (Atapuerca) compared with those of extinct and extant specimens. Topographic thickness variation is rendered by a pseudo-colour scale ranging from thinner (dark-blue) to thicker (red). EAH = East African Homo from Eritrea (MA 93), NEA = Neanderthal (La Chaise de Vouthon S14-7) and MH = modern human of European origin (o = occlusal, b = buccal). Scale bar = 1.95 for all specimens.

**Fig 10:**
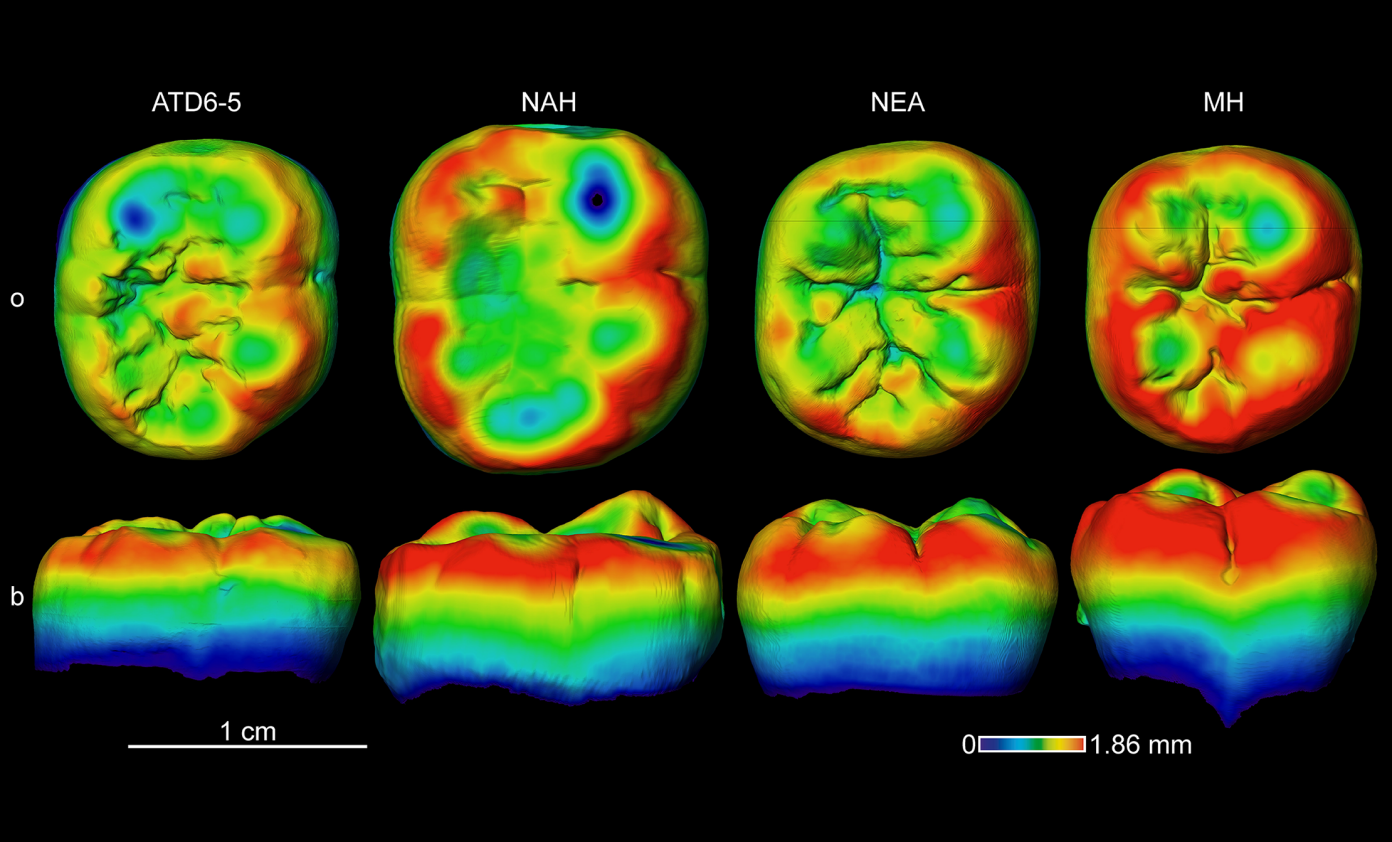
Enamel thickness cartographies of the *H*. *antecessor* lower M2 (ATD6-5) from Gran Dolina (Atapuerca) compared with those of extinct and extant specimens. Topographic thickness variation is rendered by a pseudo-colour scale ranging from thinner (dark-blue) to thicker (red). NAH = North African Homo from Tighenif (Tighenif2), NEA = Neanderthal (Krapina D10) and MH = modern human of European origin (o = occlusal, b = buccal). Scale bar = 1.86 for all specimens.

**Fig 11:**
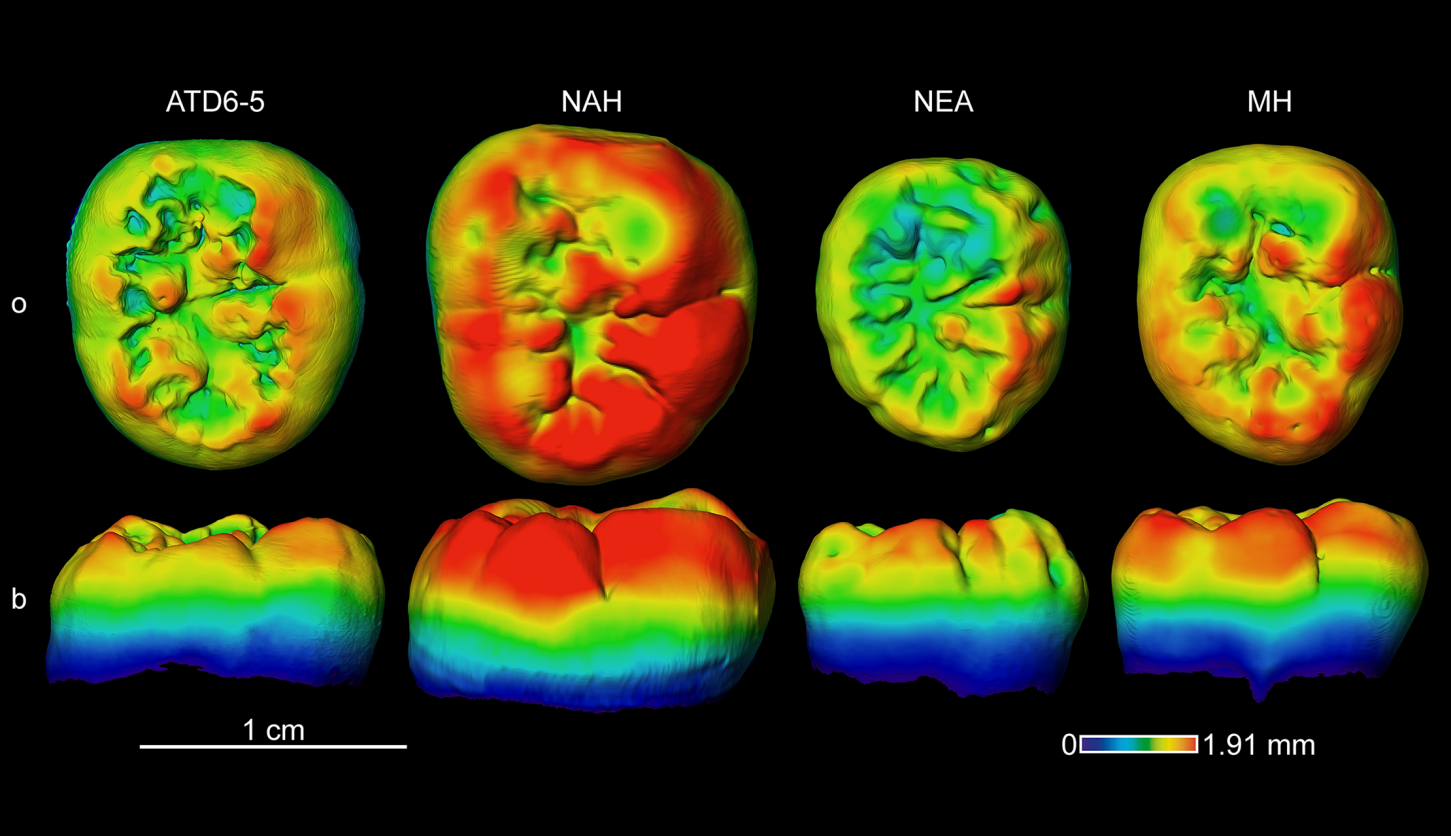
Enamel thickness cartographies of the *H*. *antecessor* lower M3 (ATD6-5) from Gran Dolina (Atapuerca) compared with those of extinct and extant specimens. Topographic thickness variation is rendered by a pseudo-colour scale ranging from thinner (dark-blue) to thicker (red). NAH = North African Homo from Tighenif (Tighenif2), NEA = Neanderthal (La Chaise de Vouthon S43) and MH = modern human of European origin (o = occlusal, b = buccal). Scale bar = 1.91 for all specimens.

Chromatic maps of TD6 molars reflect different signal than what we described for the thickness proportions (Figs 7–11). The TD6 maxillary M1s, such as the specimen ATD6-94 illustrated in the Fig 7, approximate the Neanderthal figure in terms of the absolute thickness, but resemble both Neanderthals and modern humans in their relative distribution of enamel, with a similar pattern on the four main cusps and the lingual aspect.

Although TD6 maxillary M2 (Fig 8) share with Neanderthals and modern humans thicker enamel over lingual cusps, the overall relative pattern of distribution is more similar to that of Neanderthals.

In TD6, the mandibular M1 (Fig 9) shows thicker enamel distributed at the external periphery of the buccal cusps and along the occlusal marginal ridges. This pattern is similar to that of the Eritrean late Early Pleistocene *H*. *erectus/ergaster* specimen and to the modern human figure, but differ from the Neanderthal specimen for which the thickest enamel is more evenly spread over the occlusal area.

However, when looking at the mandibular M2 and M3 (Figs 10 and 11), the TD6 specimens more closely approximate the Neanderthal pattern, than the more spread thick enamel of the North African late Early-early Middle specimen from Tighenif and the modern humans.

## Discussion

Dental studies of the outer and inner structures identified a derived pattern characteristic of the European Neanderthal lineage [8, 10, 13–26, 48, 50, 51, 68, 69]. Even if enamel thickness is no longer regarded as a useful trait to assess phylogenetic relationships in the genus *Homo* or at least at a large scale [10], the possible existence of local chronogeographic trends still needs to be assessed. In fact, tooth tissue proportions, including enamel thickness indices and distribution maps are reliable indicators of the Neanderthal signal [8, 10, 17, 20, 55]. However, the chronological and geographic context of appearance of this typical dental endostructural condition remains unknown. Therefore, the exploration of the internal structural organization of European Early and Middle Pleistocene hominin groups is relevant to explore this question. In particular, the molar assemblage belonging to *H*. *antecessor*, chronologically compatible with the timing of divergence of Neanderthals and modern humans [41], could contribute to narrow the context for the appearance of this condition.

The TD6 permanent molars exhibit thick average and relative enamel. This condition, regarded as the primitive trait [1, 7, 8, 70, 71], is shared with the majority of hominin species [1, 8, 10–12, 54] except for Neanderthals [8, 10, 55, 62] and some isolated specimens, such as African Early Pleistocene specimen from Eritrea [56]. Overall, TD6 molars exhibit rather low percentage of dentine in the crown and relative thick enamel in both linear and 3D estimates. *H*. *antecessor* estimated values approximate the condition documented for late Early to Middle Pleistocene hominin *H*. *erectus* specimens from Sangiran [54] and modern humans [8–10]. Moreover, for these two sets of measurements, Z-score confirmed the proximity of TD6 molars with extant human populations, rather than with Neanderthals. According to developmental studies, thick enamel in hominins can result from different developmental mechanisms [5, 30]. Dean and colleagues [29] observed that thick enamel in *H*. *sapiens* is controlled by a unique odontogenetic process, different from that of *Australopithecus* and other species of the genus *Homo*. For *H*. *sapiens*, Smith and colleagues [10] suggested that generalized dental reduction was behind the decrease in coronal dentine in comparison with enamel, leading to the relative thick enamel characteristic of this species. However, interestingly during the Pleistocene-Holocene transition, preliminary results suggest that in the process of dental reduction, the volume of enamel decreased in comparison to the dentine [72]. In this way, we could relate *H*. *antecessor* thick enamel condition to a series of processes. Studies about dental development in *H*. *antecessor* already identified a shift in the pattern of development for this species in relation to early species. Bermúdez de Castro et al. [10] concluded that *H*. *antecessor* (specimen ATD6-112 mandibular fragment with M1) possessed a modern-like pattern of dental development that differed from that of early African and Asian species. Moreover, the significant size reduction in the post-canine dentition of *H*. *antecessor* in comparison to early African and Asian *Homo* species [46] could explain the reduced coronal dentine. As suggested for *H*. *sapiens*, in TD6 hominins the size reduction in the posterior dentition could result in a decreased quantity of coronal dentine. Moreover, the reduction of the crown dentine in genus *Homo* was also accompanied by the reduction of the root dentine and mandibular bones [73, 74]. Studies have suggested similarities between dentine and bone tissue to explain this reduction ([10, 75] and references therein). Estimates of body proportions in *H*. *antecessor* based on the metrics of the talus (specimen ATD6-95 and associated, based on size similarities, to the mandibular fragment ATD6-113) calculated a stature and body mass of approximately 173cm and 76kg for this male individual [76, 77]. Analyses of TD6 postcranial remains, including the talus, clavicle, radius and humerus (specimens ATD6-95, ATD6-50, ATD6-43 and ATD6-148, respectively) showed that TD6 individuals presented similar stature and build to the male individuals from the Middle Plesitocene site of Atapuerca-Sima de los Huesos (SH), an indicator of the species robusticity [43, 44, 76, 77]. If future studies confirm these results, we could advance that the reduction in dental size and, in particular, the extreme dentine reduction in *H*. *antecessor* is not linked to skeletal gracility, but to an independent biological process.

Neanderthal thin AET and RET result from larger dentine proportions characterised by a complex topography and larger surface of the EDJ [8, 25]. Unlike Neanderthals [7, 78], *H*. *antecessor* revealed low mandibular dentine horns [79], the ancestral trait for the *Homo* clade. This characteristic also contributes to explain the thick enamel condition observed in TD6 specimens. When only the lateral enamel thickness is considered, the TD6 molars estimates reveal a different pattern, with globally higher values of lateral percentage of dentine compared to the fossil and extant samples. Even if the TD6 estimates are comparable to those of Neanderthals. For the LRET, the TD6 molar estimates overlap with the extinct and extant samples. However, if we exclude the specimen ATD6-96 (M1-M3) from the analysis, TD6 LRET approximates the Neanderthal condition. This difference in the results is the consequence of the unusual circular morphology of the crowns associated to the reduced dimensions in ATD6-69 molars. This morphology is also responsible for the peculiar aspect of the internal structural organization such as the extremely low dentine horns, and lack of occlusal crenulations and crests [80] probably affecting the tissue proportions. In any case, Z-score analyses confirmed the tendency of the TD6 molars to shift slightly towards the Neanderthal condition than to the modern human condition. In this way, the results presented here further suggest the contribution of the occlusal surface to the thick relative enamel condition in *H*. *antecessor* and the more Neanderthal-like signal recorded in the cervical region of TD6 molars. Finally, as described for the lateral enamel thickness, the chromatic maps evinced the proximity between the TD6 molars and Neanderthals in terms of the relative pattern of enamel distribution among the crown.

## Conclusions

In this study, we provided for the first time data about dental tissue proportions and enamel thickness in the Early Pleistocene *H*. *antecessor* molars, and contribute to the knowledge of the variability of this trait in the genus *Homo*. *H*. *antecessor* molars tend to show on average 2D and 3D (complete crown and lateral enamel) thicker absolute and relative enamel, approximating TD6 molars to the majority of the fossil sample and modern human condition, and in contrast to the Neanderthal condition. However, the proportion of tissues among the crown is variable. Compared to Neanderthals and modern humans, TD6 molars present the lowest values of crown percentage of dentine. On the contrary, on the lateral aspect of the crown the values of percentage of dentine are high, resembling the Neanderthal signal, and highlighting the contribution of the occlusal surface to the thick condition. Similarly, the *H*. *antecessor* molar enamel distribution maps reveal a relative distribution pattern that is more similar to the Neanderthal condition (with the thickest enamel more spread at the periphery of the occlusal basin) rather than that of modern humans (with thicker cuspal enamel). Together with the lateral tissue proportions, the relative pattern of enamel distribution among the TD6 molar crowns also resembles the Neanderthal condition. Future studies on European Middle Pleistocene populations, such as Atapuerca-SH, will provide a more comprehensive understanding on the variability of this trait in this important period, the transition from the Early to the Middle Pleistocene in Europe.

## Supporting information

S1 Fig Reconstruction of the worn molar cap. Reconstruction of TD6 worn molar cap by superimposition of an unworn molar cap.
(TIF)

S1 Table 2D values measured in the TD6 maxillary and mandibular molars and those of the extinct and extant specimens/populations. Upper molars: *H*. *antecessor* from Gran Dolina (original data). HER: *H*. *erectus* (Sangiran_M1, Zanolli [54]; China_M2, Smith et al. [10]; Xing et al. [49]). EMPH: European Middle Pleistocene *Homo* (Steinheim_M1, Smith et al. [10]). NAH: North African *Homo* (Thomas Quarry_M2, Smith et al. [10]). NEA: Neanderthals (Olejniczak et al. [8]). FHS: fossil *H*. *sapiens* (Qafzeh_M2, Smith et al. [10]). MH: modern humans (Smith et al. [9, 10]and *pers*. *comm*.). Lower molars: *H*. *antecessor* from Gran Dolina (original data). EAH: East African *Homo* (Eritrea_M1, Zanolli et al. [56]). NAH: North African *Homo* (Tighenif_M2, Zanolli and Mazurier [11]). HER: *H*. *erectus* (Sangiran_M2 & M3; Zanolli [54]). EMPH: European Middle Pleistocene *Homo* (Mauer_M3, Smith et al. [10]. EMPH_BH: European Middle Pleistocene *Homo* (Mala Balanica_M3, Skinner et al. [24]). NEA: Neanderthals (Olejniczak et al.[8]). MH: modern humans (Smith et al. [9, 10] and Smith *pers*. *comm*.).
(DOCX)

S2 Table 3D enamel thickness values measured in the TD6 maxillary and mandibular molars and those of the extinct and extant specimens/populations. Upper molars: *H*. *antecessor* from Gran Dolina (original data). HER: *H*. *erectus* (Sangiran_M1, Zanolli [54]). NEA: Neanderthals (Olejniczak et al.[8]; Bayle et al. [55]. MH: modern humans (Olejniczak et al. [8]). Lower molars: *H*. *antecessor* from Gran Dolina (original data). EAH: East African *Homo* (Eritrea_M1, Zanolli et al. [56]). NAH: North African *Homo* (Tighenif_M2&M3, Zanolli and Mazurier [11]). HER: *H*. *erectus* (Sangiran, Zanolli [54]). NEA: Neanderthals (Olejniczak et al. [8]). MH: modern humans (Olejniczak et al. [8]; Weber and Bookstein [57] and original data).
(DOCX)

S3 Table 3D lateral enamel thickness values measured in the TD6 maxillary and mandibular molars and those of the extinct and extant specimens/populations. Upper molars: *H*. *antecessor* from Gran Dolina (original data). NAH: North African *Homo* (Tighenif_M2&M3, Zanolli and Mazurier [11]). EMPH: European Middle Pleistocene *Homo* (Visogliano6_M1 & Visogliano3_M2, Zanolli et al. [17]). NEA: Neanderthals (original data). MH: modern humans (European origin, original data). Lower molars: *H*. *antecessor* from Gran Dolina (original data). NAH: North African *Homo* (Tighenif_M1&M3; Zanolli and Mazurier [11]). HER: *H*. *erectus* (Sangiran_M1&M2; HER: *H*. *erectus* (Sangiran, Zanolli [54]). EMPH: European Middle Pleistocene *Homo* (Fontana Ranuccio_M1, Zanolli et al. [17]).NEA: Neanderthals (original data). MH: modern humans (European origin, original data and Weber and Bookstein [57]).
(DOCX)
