# Supplementary figures and images for: Tooth crown tissue proportions and enamel thickness in Early Pleistocene Homo antecessor molars (Atapuerca, Spain)
Source: PLoS One. 2018 Oct 3;13(10):e0203334. doi: 10.1371/journal.pone.0203334 (PMC6169863; doi:10.1371/journal.pone.0203334)

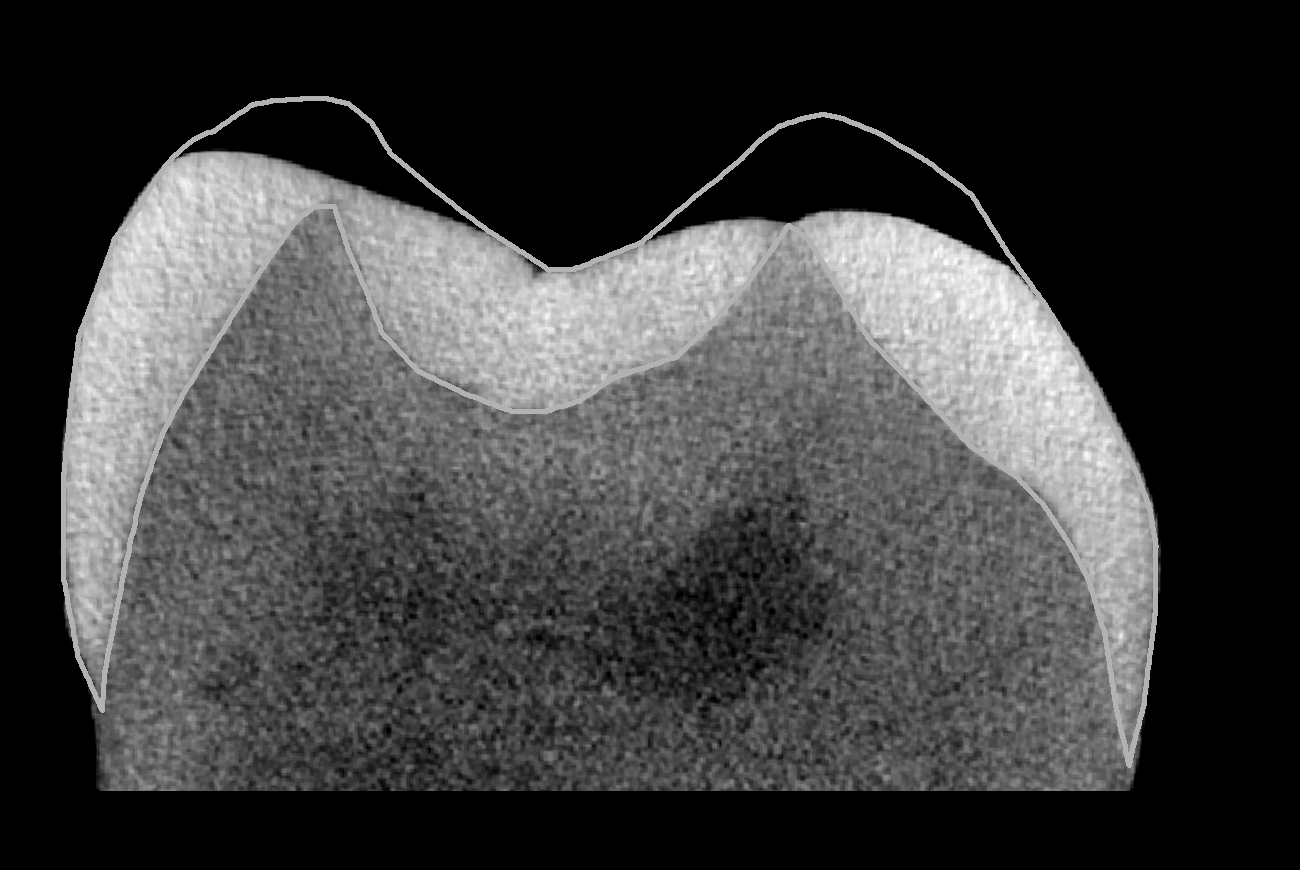

Supplement: S1 Fig — Reconstruction of TD6 worn molar cap by superimposition of an unworn molar cap. (TIF) [file pone.0203334.s001.tif]
