# Supplementary material for: Tooth crown tissue proportions and enamel thickness in Early Pleistocene Homo antecessor molars (Atapuerca, Spain)
Source: PLoS One. 2018 Oct 3;13(10):e0203334. doi: 10.1371/journal.pone.0203334 (PMC6169863; doi:10.1371/journal.pone.0203334)
Supplement: S1 Table — Upper molars: H. antecessor from Gran Dolina (original data). HER: H. erectus (Sangiran_M1, Zanolli [54]; China_M2, Smith et al. [10]; Xing et al. [49]). EMPH: European Middle Pleistocene Homo (Steinheim_M1, Smith et al. [10]). NAH: North African Homo (Thomas Quarry_M2, Smith et al. [10]). NEA: Neanderthals (Olejniczak et al. [8]). FHS: fossil H. sapiens (Qafzeh_M2, Smith et al. [10]). MH: modern humans (Smith et al. [9, 10]and pers. comm.). Lower molars: H. antecessor from Gran Dolina (original data). EAH: East African Homo (Eritrea_M1, Zanolli et al. [56]). NAH: North African Homo (Tighenif_M2, Zanolli and Mazurier [11]). HER: H. erectus (Sangiran_M2 & M3; Zanolli [54]). EMPH: European Middle Pleistocene Homo (Mauer_M3, Smith et al. [10]. EMPH_BH: European Middle Pleistocene Homo (Mala Balanica_M3, Skinner et al. [24]). NEA: Neanderthals (Olejniczak et al.[8]). MH: modern humans (Smith et al. [9, 10] and Smith pers. comm.). (DOCX) [file pone.0203334.s002.docx]

S1 Table. 2D values measured in the TD6 maxillary and mandibular molars and those of the extinct and extant specimens/populations

| **Taxon/group** | **Specimen** | **Tooth** | **Wear** | **c (mm^2^)** | **b (mm^2^)** | **a (mm^2^)** | **e (mm)** | **Acdp/Ac (%)** | **AET (mm)** | **RET** | **Data Source** |  |
| --- | --- | --- | --- | --- | --- | --- | --- | --- | --- | --- | --- | --- |
| TD6 | AT6-10 | UM1 | 3 | 20.52 | 43.04 | 63.56 | 19.24 | 67.71 | 1.06 | 16.25 | Original data |  |
|  | AT6-11 |  | 3 | 24.23 | 43.17 | 67.40 | 20.06 | 64.05 | 1.20 | 18.38 |  |  |
|  | AT6-69 |  | 2 | 22.72 | 37.33 | 60.05 | 20.84 | 62.16 | 1.09 | 17.84 |  |  |
|  | AT6-103 |  | 2 | 24.56 | 47.66 | 72.22 | 22.14 | 65.99 | 1.10 | 16.06 |  |  |
| Mean |  |  |  | 23.01 | 42.80 | 65.81 | 20.57 | 64.98 | 1.12 | 17.14 |  |  |
| SD |  |  |  | 1.84 | 4.23 | 5.22 | 1.23 | 2.40 | 0.06 | 1.15 |  |  |
| Range |  |  |  | 20.52-23.01 | 37.33-47.66 | 60.05-72.22 | 19.24-22.14 | 62.14-67.71 |  |  |  |  |
| HER | NG91-G10nº1 |  |  | 28.9 | 46.60 | 75.50 | 22.2 | 61.72 | 1.30 | 19.07 | Zanolli [54] |  |
| EMPH | Steinheim |  |  | 23.25 | 40.99 | 64.24 | 21.49 | 63.80 | 1.08 | 16.89 | Smith et al. [10] |  |
| NEA | Engis |  |  | 23.29 | 43.85 | 67.14 | 22.88 | 65.31 | 1.01 | 15.37 | Olejniczak et al.[8] |  |
|  | SR1105 |  |  | 28 | 49.75 | 77.75 | 23.45 | 63.98 | 1.19 | 16.92 |  |  |
|  | Le Moustier1 |  |  | 21.44 | 45.64 | 67.08 | 23 | 68.03 | 0.93 | 13.79 |  |  |
|  | SCLA_4A_4 |  |  | 21.13 | 43.27 | 64.40 | 21.38 | 67.18 | 0.98 | 15.02 |  |  |
|  | La Quina |  |  | 21.03 | 37.19 | 58.22 | 21.08 | 63.87 | 0.99 | 16.35 | Original data |  |
| Mean |  |  |  | 22.97 | 43.94 | 66.918 | 22.35 | 65.68 | 1.02 | 15.49 |  |  |
| SD |  |  |  | 2.95 | 4.54 | 7.06 | 1.05 | 1.87 | 0.099 | 1.21 |  |  |
| Range |  |  |  | 21.03-28.0 | 37.19-49.75 | 58.22-77.75 | 21.08-23.45 | 63.87-68.03 | 0.93-1.19 | 13.79-16.92 |  |  |
| MH | 37 specimens |  |  |  |  |  |  |  |  |  |  |  |
| Mean |  |  |  | 42.87 | 25.18 | 68.05 | 20.64 | 62.85 | 1.22 | 18.75 | Smith et al. [9]  Smith *pers. comm.* |  |
| SD |  |  |  | 6.25 | 3.16 |  | 1.51 | 0.02 | 0.12 | 2.08 |  |  |
| Range |  |  |  | 32.46-59.44 | 20.05-31.82 |  | 17.66-24.11 | 57.16-68.98 | 0.98-1.50 | 13.95-23.86 |  |  |
| TD6 | ATD6-12 | UM2 | 2 | 23.99 | 39.05 | 63.04 | 18.95 | 61.94 | 1.26 | 20.25 | Original data |  |
|  | ATD6-69 |  | 1 | 29.33 | 42.83 | 72.16 | 19.30 | 59.35 | 1.51 | 23.22 | Original data |  |
| Mean |  |  |  | 26.66 | 40.94 | 67.60 | 19.13 | 60.65 | 1.39 | 21.74 |  |  |
| SD |  |  |  | 3.77 | 2.67 | 6.44 | 0.24 | 1.83 | 0.17 | 2.09 |  |  |
| HER | CA 771 |  |  | 33.73 | 57.70 | 91.43 | 22.86 | 63.10 | 1.47550306 | 19.4246056 | Smith et al. [10] |  |
| HER | PA833 |  |  |  |  |  |  |  | 1.51 | 23.52 | Xing et al. [50] |  |
| NAH | Thomas Quarry |  |  | 32.02 | 56.78 | 88.80 | 22.59 | 63.9414414 | 1.41744135 | 18.8108059 | Smith et al. [10] |  |
| EMPH | Steinheim |  |  | 25.79 | 48.99 | 74.78 | 21.51 | 65.512169 | 1.19897722 | 17.129994 | Smith et al. [10] |  |
| NEA | SR332 |  |  | 26.6 | 38.55 | 65.15 | 20.55 | 59.17 | 1.29 | 20.84 | Olejniczak et al. [8] |  |
|  | SR4 |  |  | 29.05 | 58.72 | 87.77 | 24.22 | 66.90 | 1.19 | 15.65 |  |  |
|  | SR531 |  |  | 23.24 | 39.35 | 62.59 | 20.6 | 62.86 | 1.12 | 17.98 |  |  |
|  | SR551 |  |  | 28.43 | 43.79 | 72.22 | 22.05 | 60.63 | 1.28 | 19.48 |  |  |
|  | Le Moustier1 |  |  | 25.89 | 46.16 | 72.05 | 22.47 | 64.06 | 1.15 | 16.95 |  |  |
|  | SCLA_4A_3 |  |  | 22.72 | 40.58 | 63.30 | 20.03 | 64.10 | 1.13 | 17.80 |  |  |
|  | La Quina |  |  | 25.77 | 36.74 | 62.51 | 19.85 | 58.77 | 1.29 | 21.41 | Original data |  |
| Mean |  |  |  | 25.95 | 43.41 | 69.37 | 21.39 | 62.36 | 1.21 | 18.59 |  |  |
| SD |  |  |  | 2.38 | 7.46 | 9.14 | 1.58 | 2.96 | 0.07 | 2.08 |  |  |
| Range |  |  |  | 22.72-29.05 | 36.74-58.72 | 62.51-87.77 | 19.85-24.22 | 58.77-66.90 | 1.12-1.29 | 15.65-21.41 |  |  |
| FHS | Qafzeh |  |  | 25.97 | 43.63 | 69.60 | 19.82 | 62.6867816 | 1.31029263 | 19.8369863 | Smith et al. [10] |  |
| MH | 25 specimens |  |  |  |  |  |  |  |  |  | Smith et al. [9] |  |
| Mean |  |  |  | 42.76 | 28.61 | 71.37 | 20.49 | 60.05 | 1.4 | 21.59 | Smith *pers. comm.* |  |
| SD |  |  |  | 7.9 | 4.07 |  | 1.67 |  | 0.17 | 3.13 |  |  |
| Range |  |  |  | 30.12-65.71 | 23.12-36.64 |  | 18.22-24.83 | 53.80-66.45 | 1.13-1.76 | 16.49-28.03 |  |  |
| **Taxon/group** | **Specimen** | **Tooth** | **Wear** | **c (mm^2^)** | **b (mm^2^)** | **a (mm^2^)** | **e (mm)** | **Acdp/Ac (%)** | **AET (mm)** | **RET** | **Data Source** |  |
| TD6 | ATD6-5 | LM1 | 3 | 25.02 | 35.52 | 60.54 | 18.16 | 58.67 | 1.38 | 23.12 | Original data |  |
|  | ATD6-94 |  | 2 | 23.12 | 44.67 | 67.79 | 20.47 | 65.89 | 1.13 | 16.90 |  |  |
|  | ATD6-112 |  | 1 | 21.98 | 27.35 | 49.33 | 18.82 | 55.44 | 1.17 | 22.33 |  |  |
|  | ATD6-96 |  | 3 | 18.07 | 33.52 | 51.59 | 18.00 | 64.97 | 1.00 | 17.34 |  |  |
| Mean |  |  |  | 22.05 | 35.27 | 57.31 | 18.86 | 61.25 | 1.17 | 19.92 |  |  |
| SD |  |  |  | 2.93 | 7.17 | 8.50 | 1.13 | 5.03 | 0.16 | 3.26 |  |  |
| Range |  |  |  | 18.07-25.02 | 27.35-44.67 | 49.33-67.79 | 18.00-20.47 | 55.44-65.89 | 1.00-1.38 | 16.90-23.12 |  |  |
| EAH | MA 93 |  |  | 18.60 | 38.20 | 56.80 | 18.60 | 67.25 | 1.00 | 16.18 | Zanolli et al. [56] |  |
| NEA | BDJ4C |  |  | 19.56 | 40.17 | 59.73 | 19.40 | 67.25 | 1.01 | 15.91 | Olejniczak et al. [8] |  |
|  | S5 |  |  | 22.36 | 41.83 | 64.19 | 22.48 | 65.17 | 0.99 | 15.38 |  |  |
|  | LM_1_ |  |  | 22.15 | 40.18 | 62.33 | 22.56 | 64.46 | 0.98 | 15.49 |  |  |
|  | Engis |  |  | 22.10 | 43.01 | 65.11 | 22.52 | 66.06 | 0.98 | 14.96 |  |  |
|  | G-1048-69 |  |  | 21.06 | 38.01 | 59.07 | 20.50 | 64.35 | 1.03 | 16.66 |  |  |
|  | SR755 |  |  | 22.07 | 43.91 | 65.98 | 20.27 | 66.55 | 1.09 | 16.43 |  |  |
|  | SR540 |  |  | 21.04 | 33.38 | 54.42 | 17.80 | 61.34 | 1.18 | 20.46 |  |  |
|  | 1 |  |  | 21.20 | 43.42 | 64.62 | 21.54 | 67.19 | 0.98 | 14.94 |  |  |
|  | 1 |  |  | 16.82 | 34.60 | 51.42 | 17.65 | 67.29 | 0.95 | 16.20 |  |  |
|  | SCLA_4A_1 |  |  | 20.01 | 45.12 | 65.13 | 21.64 | 69.28 | 0.92 | 13.77 |  |  |
|  | S49 |  |  | 20.65 | 45.42 | 66.07 | 22.25 | 68.75 | 0.93 | 13.77 |  |  |
|  | S14-7 |  |  | 21.78 | 40.18 | 61.96 | 22.11 | 64.85 | 0.99 | 15.54 |  |  |
|  |  |  |  | 23.81 | 36.45 | 60.26 | 23.25 | 60.49 | 1.02 | 16.96 |  |  |
| Mean |  |  |  | 21.12 | 40.44 | 61.56 | 21.07 | 65.62 | 1.00 | 15.88 |  |  |
| SD |  |  |  | 1.70 | 3.90 | 4.54 | 1.83 | 2.59 | 0.07 | 1.69 |  |  |
| Range |  |  |  | 16.82-23.81 | 33.38-45.42 | 51.42-65.98 | 17.65-23.25 | 60.48-69.27 | 0.92-1.18 | 13.76-20.45 |  |  |
| MH | 55 specimens |  |  |  |  |  |  |  |  |  |  |  |
| Mean |  |  |  | 40.16 | 21.74 | 61.90 | 20.32 | 64.48 | 1.07 | 16.99 | Smith et al. [9]  Smith *pers. comm* |  |
| SD |  |  |  | 5.02 | 2.95 |  | 1.28 | 1.17 | 0.13 | 2.29 |  |  |
| Range |  |  |  | 27.45-50.82 | 16.21-28.58 |  | 16.73-22.94 | 59.19-72.65 | 0.80-1.40 | 11.76-22.62 |  |  |
| TD6 | AT6-5 | LM2 | 2 | 17.40 | 33.74 | 51.14 | 16.97 | 65.98 | 1.03 | 17.65 | Original data | |
|  |  |  |  |  |  |  |  |  |  |  |  | |
|  | AT6-144 |  | 1 | 22.98 | 25.43 | 48.41 | 16.72 | 52.53 | 1.37 | 27.25 |  | |
|  | ATD6-96 |  | 2 | 16.34 | 25.14 | 41.48 | 16.03 | 60.61 | 1.02 | 20.33 |  | |
| Mean |  |  |  | 19.51 | 29.38 | 48.89 | 16.80 | 60.01 | 1.16 | 21.60 |  | |
| SD |  |  |  | 2.73 | 4.10 | 4.80 | 0.52 | 4.81 | 0.15 | 3.51 |  | |
|  |  |  |  | 16.34-22.98 | 25.14-33.74 | 41.48-54.54 | 16.03-16.97 | 52.53-65.98 | 1.002-1.37 | 17.65-27.25 |  | |
| NAH | Tighenif 2 |  |  | 25.20 | 47.70 | 72.90 | 21.10 | 65.43 | 1.19 | 17.29 | Zanolli and Mazurier  [11] | |
| HER | NG0802.3 |  |  | 21.20 | 31.20 | 52.40 | 17.40 | 59.54 | 1.22 | 21.81 | Zanolli [54] | |
|  | NG92.3 |  |  | 23.40 | 31.50 | 54.90 | 17.90 | 57.38 | 1.31 | 23.29 |  | |
|  | NG92D6ZE57s/d76 | |  | 24.10 | 41.30 | 65.40 | 20.20 | 63.15 | 1.19 | 18.56 |  |  |
|  | NG0802.2 |  |  | 24.00 | 30.60 | 54.60 | 17.40 | 56.04 | 1.38 | 24.93 |  | |
| Mean |  |  |  | 23.18 | 33.65 | 56.83 | 18.23 | 59.03 | 1.27 | 22.15 |  | |
| Range |  |  |  | 21.2-23.4 | 30.60-41.30 | 52.40-65.40 | 17.40-20.20 | 56.04-59.54 | 1.19-1.37 | 18.56-24.93 |  | |
| NEA | S36 |  |  | 17.10 | 33.85 | 50.95 | 17.78 | 66.44 | 0.96 | 16.53 | Olejniczak et al. [8] | |
|  | KRD6 |  |  | 22.43 | 47.56 | 69.99 | 22.89 | 67.95 | 0.98 | 14.21 |  | |
|  | 1 |  |  | 24.62 | 46.28 | 70.90 | 21.54 | 65.28 | 1.14 | 16.80 |  | |
|  | 1 |  |  | 19.06 | 39.81 | 58.87 | 18.73 | 67.62 | 1.02 | 16.13 |  | |
|  | 1 |  |  | 17.22 | 39.60 | 56.82 | 18.29 | 69.69 | 0.94 | 14.96 |  | |
|  | SCLA_4A_1 |  |  | 22.24 | 44.33 | 66.57 | 20.73 | 66.59 | 1.07 | 16.11 |  | |
|  | KRD10 |  |  | 22.53 | 45.19 | 67.72 | 21.08 | 66.73 | 1.07 | 15.90 |  | |
|  | KRD10 |  |  | 22.53 | 45.19 | 67.72 | 22.89 | 66.73 | 0.98 | 14.64 |  | |
| Mean |  |  |  | 20.97 | 42.73 | 63.69 | 20.49 | 67.13 | 1.02 | 15.66 |  | |
| SD |  |  |  | 2.79 | 4.59 | 7.22 | 2.01 | 1.31 | 0.07 | 0.94 |  | |
| Range |  |  |  | 17.1-22.53 | 33.85-47.56 | 50.95-70.90 | 17.78-22.89 | 65.27-67.95 | 0.94-1.14 | 14.2016.80 |  | |
| MH | 45 specimens |  |  |  |  |  |  |  |  |  | Smith et al. [9]  Smith *pers. comm* | |
| Mean |  |  |  | 34.33 | 22.05 | 56.38 | 18.52 | 60.78 | 1.19 | 20.51 |  | |
| SD |  |  |  | 4.26 | 2.59 |  | 1.24 | 0.03 | 0.14 | 2.93 |  | |
| Range |  |  |  | 23.75-42.24 | 16.81-29.13 |  | 15.22-21.60 | 53.21-67.80 | 0.94-1.55 | 14.85-27.66 |  | |
| TD6 | AT6-5 | LM3 | 1 | 20.00 | 30.65 | 50.65 | 16.68 | 60.51 | 1.20 | 21.66 | Original data | |
|  | AT6-113 |  | 2 | 17.83 | 28.45 | 46.28 | 15.80 | 61.47 | 1.13 | 21.16 |  | |
|  | ATD6-96 |  | 1 | 12.72 | 13.97 | 26.69 | 11.66 | 52.34 | 1.09 | 29.19 |  | |
| Mean |  |  |  | 16.85 | 24.36 | 41.21 | 14.71 | 58.11 | 1.14 | 24.00 |  | |
| SD |  |  |  | 3.05 | 7.40 | 10.42 | 2.19 | 4.10 | 0.04 | 3.67 |  | |
| Range |  |  |  | 12.72-20.00 | 13.97-30.65 | 26.69-50.65 | 11.66-16.68 | 52.33-61.47 | 1.09-1.20 | 29.19-21.16 |  | |
| HER | NG9107.2 |  |  | 15.90 | 24.10 | 40.00 | 16.30 | 60.25 | 0.98 | 19.87 | Zanolli [54] | |
| EMPH | Mauer |  |  |  |  |  |  |  | 1.27 | 21.60 | Smith et al. [10] | |
|  | BH-1 |  |  | 15.22 | 27.48 | 42.70 | 16.26 | 64.36 | 0.94 | 17.86 | Skinner et al. [24] | |
| NEA | S36 |  |  | 18.17 | 34.70 | 52.87 | 18.11 | 65.63 | 1.00 | 17.03 | Olejniczak et al. [8] | |
|  | KRD9 |  |  | 19.56 | 42.92 | 62.48 | 19.57 | 68.69 | 1.00 | 15.26 |  | |
|  | 1 |  |  | 22.00 | 40.16 | 62.16 | 19.91 | 64.61 | 1.10 | 17.44 |  | |
|  | 1 |  |  | 24.84 | 38.03 | 62.87 | 21.35 | 60.49 | 1.16 | 18.87 |  | |
|  | 1 |  |  | 16.29 | 32.25 | 48.54 | 17.75 | 66.44 | 0.92 | 16.16 |  | |
|  | 1 |  |  | 17.36 | 32.76 | 50.12 | 17.62 | 65.36 | 0.99 | 17.21 |  | |
|  | Q760-H9 |  |  | 20.74 | 48.02 | 68.76 | 20.93 | 69.84 | 0.99 | 14.30 |  | |
|  | BD01 |  |  | 16.70 | 30.58 | 47.28 | 18.14 | 64.68 | 0.92 | 16.65 |  | |
|  | S43 |  |  | 17.90 | 30.93 | 48.83 | 17.56 | 63.34 | 1.02 | 18.33 |  | |
| Mean |  |  |  | 19.28 | 36.71 | 55.99 | 18.99 | 65.45 | 1.01 | 16.80 |  | |
| SD |  |  |  | 2.80 | 6.03 | 8.04 | 1.48 | 2.76 | 0.08 | 1.43 |  | |
| Range |  |  |  | 16.70-24.84 | 30.58-48.02 | 47.28-68.76 | 17.56-20.93 | 63.34-69.83 | 0.91-1.16 | 14.29-18.86 |  | |
|  |  |  |  |  |  |  |  |  |  |  |  | |
| MH | 44 specimens |  |  |  |  |  |  |  |  |  | Smith et al. [9]  Smith *pers. comm* | |
| Mean |  |  |  | 33.09 | 22.58 | 55.67 | 18.27 | 59.31 | 1.24 | 21.63 |  | |
| SD |  |  |  | 5.11 | 3.28 |  | 1.36 | 0.03 | 0.15 | 2.99 |  | |
| Range |  |  |  | 24.40-45.98 | 16.75-29.42 |  | 15.90-22.26 | 50.82-64.61 | 0.98-1.67 | 17.22-31.84 |  | |

Upper molars: *H. antecessor* from Gran Dolina (original data). HER: *H. erectus* (Sangiran_M1, Zanolli [54]; China_M2, Smith et al. [10] ; Xing et al. [49]). EMPH: European Middle Pleistocene *Homo* (Steinheim_M1, Smith et al. [10]). NAH: North African *Homo* (Thomas Quarry_M2, Smith et al. [10]). NEA: Neanderthals (Olejniczak et al. [8]). FHS: fossil *H. sapiens* (Qafzeh_M2, Smith et al. [10]). MH: modern humans (Smith et al. [9, 10]and *pers. comm*.). Lower molars: *H. antecessor* from Gran Dolina (original data). EAH: East African *Homo* (Eritrea_M1, Zanolli et al. [56]). NAH: North African *Homo* (Tighenif_M2, Zanolli and Mazurier [11]). HER: *H. erectus* (Sangiran_M2 & M3; Zanolli [54] ). EMPH: European Middle Pleistocene *Homo* (Mauer_M3, Smith et al. [10]. EMPH_BH: European Middle Pleistocene *Homo* (Mala Balanica_M3, Skinner et al. [24]). NEA: Neanderthals (Olejniczak et al.[8]). MH: modern humans (Smith et al. [9, 10] and Smith *pers. comm*.).
