# Supplementary material for: Tooth crown tissue proportions and enamel thickness in Early Pleistocene Homo antecessor molars (Atapuerca, Spain)
Source: PLoS One. 2018 Oct 3;13(10):e0203334. doi: 10.1371/journal.pone.0203334 (PMC6169863; doi:10.1371/journal.pone.0203334)
Supplement: S2 Table — Upper molars: H. antecessor from Gran Dolina (original data). HER: H. erectus (Sangiran_M1, Zanolli [54]). NEA: Neanderthals (Olejniczak et al.[8]; Bayle et al. [55]. MH: modern humans (Olejniczak et al. [8]). Lower molars: H. antecessor from Gran Dolina (original data). EAH: East African Homo (Eritrea_M1, Zanolli et al. [56]). NAH: North African Homo (Tighenif_M2&M3, Zanolli and Mazurier [11]). HER: H. erectus (Sangiran, Zanolli [54]). NEA: Neanderthals (Olejniczak et al. [8]). MH: modern humans (Olejniczak et al. [8]; Weber and Bookstein [57] and original data). (DOCX) [file pone.0203334.s003.docx]

S2 Table. 3D enamel thickness values measured in the TD6 maxillary and mandibular molars and those of the extinct and extant specimens/populations

| **Taxon/group** | **Specimen** | **Tooth** | **Wear** | **Ve (mm^3^)** | **Vcdp (mm^3^)** | **Vc (mm^3^)** | **SEDJ (mm^2^)** | **Vcdp/Vc (%)** | **3D AET (mm)** | **3D RET** | **Data Source** |
| --- | --- | --- | --- | --- | --- | --- | --- | --- | --- | --- | --- |
| *H. antecessor* | AT6-69 | UM1 | 2 | 279.20 | 288.59 | 567.79 | 204.69 | 50.83 | 1.36 | 20.64 | Original data |
|  | AT6-103 | UM1 | 2 | 324.29 | 402.75 | 727.04 | 262.22 | 55.40 | 1.24 | 16.75 |  |
| Mean |  |  |  | 301.75 | 345.67 | 647.41 | 233.45 | 53.11 | 1.30 | 18.69 |  |
| SD |  |  |  | 31.88 | 80.73 | 112.61 | 40.68 | 3.23 | 0.09 | 2.75 |  |
| HER | NG91-G10nº1 |  |  | 275.00 | 331.60 | 606.60 | 243.80 | 54.67 | 1.13 | 16.30 | Zanolli [54] |
| NEA | Engis |  |  | 249.44 | 355.59 | 605.03 | 232.74 | 58.77 | 1.07 | 15.13 | Olejniczak et al. [8] |
|  | KRD134 |  |  | 341.92 | 460.43 | 802.35 | 317.81 | 57.38 | 1.08 | 13.93 | Original data from NESPOS database |
|  | KRD101 |  |  | 259.48 | 272.83 | 532.31 | 217.26 | 48.80 | 1.19 | 18.72 |  |
| Mean |  |  |  | 283.61 | 353.13 | 646.56 | 255.93 | 54.98 | 1.11 | 15.92 |  |
| SD |  |  |  | 50.74 | 94.01 | 139.72 | 54.13 | 5.40 | 0.06 | 2.49 |  |
| Range |  |  |  | 249.44-341.92 | 272.93-460.43 | 532.31-802.35 | 217.26-317.81 | 48.79-58.77 | 1.07-1.19 | 13.93-18.71 |  |
| MH |  |  |  | 185.59 | 285.79 | 471.38 | 163.86 | 60.63 | 1.13 | 17.19 | Olejniczak et al. [8] |
|  |  |  |  | 185.21 | 296.10 | 481.31 | 184.57 | 61.52 | 1.00 | 15.06 |  |
|  |  |  |  | 214.32 | 294.90 | 509.22 | 255.00 | 57.91 | 0.84 | 12.63 |  |
|  |  |  |  | 219.42 | 292.26 | 511.68 | 226.91 | 57.12 | 0.97 | 14.57 |  |
|  |  |  |  | 229.89 | 304.19 | 534.08 | 145.36 | 56.96 | 1.58 | 23.52 |  |
| Mean |  |  |  | 206.89 | 294.65 | 501.53 | 195.14 | 58.83 | 1.10 | 16.59 |  |
| SD |  |  |  | 20.40 | 6.66 | 25.20 | 45.17 | 2.11 | 0.29 | 4.20 |  |
| Range |  |  |  | 185.21-229.89 | 285.79-304.79 | 471.38-534.08 | 145.36-255.00 | 56.96-61.52 | 0.84-1.58 | 12.63-23.52 |  |
| *H. antecessor* | AT6-12 | UM2 | 2 | 298.30 | 334.00 | 632.30 | 226.17 | 52.82 | 1.32 | 19.01 | Original data |
|  | AT6-69 |  | 1 | 301.65 | 289.98 | 591.63 | 193.17 | 49.01 | 1.56 | 23.59 |  |
| Mean |  |  |  | 299.98 | 311.99 | 611.97 | 209.67 | 50.92 | 1.44 | 21.30 |  |
| SD |  |  |  | 2.37 | 31.13 | 28.76 | 23.33 | 2.69 | 0.17 | 3.24 |  |
| NEA | SR332 |  |  | 210.06 | 270.23 | 480.29 | 185.00 | 56.26 | 1.14 | 17.56 | Olejniczak et al. [8] |
|  | SR4 |  |  | 231.54 | 355.63 | 587.17 | 239.34 | 60.57 | 0.97 | 13.65 |  |
|  | SR531 |  |  | 255.75 | 339.28 | 595.03 | 228.35 | 57.02 | 1.12 | 16.06 |  |
|  | SR551 |  |  | 256.45 | 413.05 | 669.50 | 245.22 | 61.70 | 1.05 | 14.04 |  |
|  | Spy I |  |  | 247.87 | 418.51 | 666.38 | 250.31 | 62.80 | 0.99 | 13.24 | Bayle et al. [55] |
| Mean |  |  |  | 240.33 | 359.34 | 599.67 | 229.64 | 59.67 | 1.05 | 14.91 |  |
| SD |  |  |  | 19.67 | 60.71 | 77.06 | 26.26 | 2.89 | 0.08 | 1.84 |  |
| Range |  |  |  | 210.06-256.45 | 270.23-418.51 | 480.29-669.50 | 185.00-250.31 | 56.26-62.80 | 0.96-1.13 | 13.24-17.56 |  |
| MH |  |  |  | 188.55 | 179.58 | 368.13 | 105.67 | 48.78 | 1.78 | 31.63 | Olejniczak et al. [8] |
|  |  |  |  | 222.85 | 210.68 | 433.53 | 153.42 | 48.60 | 1.45 | 24.41 |  |
|  |  |  |  | 214.99 | 245.41 | 460.40 | 153.32 | 53.30 | 1.40 | 22.40 |  |
|  |  |  |  | 238.05 | 271.17 | 509.22 | 177.71 | 53.25 | 1.34 | 20.70 |  |
|  |  |  |  | 293.86 | 284.08 | 577.94 | 218.18 | 49.15 | 1.35 | 20.49 |  |
| Mean |  |  |  | 231.66 | 238.18 | 469.84 | 161.66 | 50.62 | 1.46 | 23.93 |  |
| SD |  |  |  | 39.13 | 43.12 | 79.04 | 41.00 | 2.44 | 0.18 | 4.59 |  |
| Range |  |  |  | 188.55-293.86 | 179.58-284.08 | 368.13-577.94 | 105.67-218.18 | 48.60-53.60 | 1.34-1.78 | 20.49-31.63 |  |
| **Taxon/group** | **Specimen** | **Tooth** | **Wear** | **Ve (mm^3^)** | **Vcdp (mm^3^)** | **Vc (mm^3^)** | **SEDJ (mm^2^)** | **Vcdp/Vc (%)** | **3D AET (mm)** | **3D RET** | **Data Source** |
| *H. antecessor* | AT6-94 | LM1 | 2 | 303.96 | 408.04 | 712.01 | 259.92 | 57.31 | 1.17 | 15.77 |  |
|  | AT6-112 |  | 1 | 282.92 | 330.75 | 613.67 | 230.67 | 53.90 | 1.23 | 17.74 |  |
| Mean |  |  |  | 293.44 | 369.40 | 662.84 | 245.29 | 55.60 | 1.20 | 16.75 |  |
| SD |  |  |  | 14.88 | 54.66 | 69.54 | 20.68 | 2.41 | 0.04 | 1.39 |  |
| EAH | MA93 |  |  | 261.40 | 332.20 | 593.60 | 256.10 | 55.96 | 1.02 | 14.74 | Zanolli et al. [56] |
|  |  |  |  |  |  |  |  |  |  |  |  |
| NEA | BDJ4C9 |  |  | 236.17 | 264.35 | 500.52 | 208.11 | 52.82 | 1.13 | 17.68 | Olejniczak et al. [8] |
|  | S14-7 |  |  | 229.87 | 332.89 | 562.76 | 231.15 | 59.15 | 0.99 | 14.35 |  |
|  | SR755 |  |  | 257.01 | 396.04 | 653.05 | 240.49 | 60.64 | 1.07 | 14.55 |  |
|  | SR540 |  |  | 257.99 | 447.75 | 705.74 | 258.84 | 63.44 | 1.00 | 13.03 |  |
|  | Engis |  |  | 234.29 | 317.58 | 551.87 | 224.59 | 57.55 | 1.04 | 15.29 |  |
|  | KRP53 |  |  | 309.52 | 429.85 | 739.37 | 302.55 | 58.14 | 1.02 | 13.56 |  |
|  | KRP54 |  |  | 221.08 | 286.44 | 507.52 | 213.49 | 56.44 | 1.04 | 15.71 |  |
|  | KRP55 |  |  | 289.05 | 422.29 | 711.34 | 268.24 | 59.37 | 1.08 | 14.36 |  |
|  | KRP D80 |  |  | 307.67 | 333.38 | 641.05 | 230.46 | 52.01 | 1.34 | 19.25 |  |
|  | RM_1_ |  |  | 202.17 | 287.88 | 490.05 | 139.71 | 58.75 | 1.45 | 21.92 |  |
|  | LM_1_ |  |  | 211.17 | 314.28 | 525.45 | 129.33 | 59.81 | 1.63 | 24.02 |  |
|  | CG IV |  |  | 189.76 | 330.38 | 520.14 | 232.74 | 63.52 | 0.82 | 11.79 |  |
| Mean |  |  |  | 245.48 | 346.93 | 592.41 | 223.31 | 58.47 | 1.13 | 16.29 |  |
| SD |  |  |  | 39.73 | 61.55 | 91.94 | 48.81 | 3.53 | 0.23 | 3.72 |  |
| Range |  |  |  | 189.76-309.52 | 246.35-447.75 | 490.05-739.37 | 129.33-302.55 | 52-63.51 | 0.81-1.63 | 11.79-24.01 |  |
| MH | Bosco Pontini 1 | |  | 246.17 | 265.03 | 511.20 | 227.50 | 51.85 | 1.08 | 16.85 | Original data |
|  | Bosco Pontini 2 | |  | 248.44 | 279.13 | 527.58 | 233.02 | 52.91 | 1.07 | 16.31 |  |
|  | San Canziano |  |  | 258.60 | 273.24 | 531.84 | 216.49 | 51.38 | 1.19 | 18.41 |  |
|  | San Canziano |  |  | 215.49 | 273.54 | 489.03 | 224.22 | 55.93 | 0.96 | 14.81 |  |
|  | Val Rosandra |  |  | 253.14 | 294.23 | 547.37 | 242.84 | 53.75 | 1.04 | 15.67 |  |
|  | B996-scht1 |  |  | 349.88 | 420.84 | 770.73 | 257.57 | 54.60 | 1.36 | 18.13 |  |
|  | B998-scht2-mand3 | |  | 241.52 | 250.21 | 491.73 | 198.48 | 50.88 | 1.22 | 19.31 |  |
|  | MH-UdP |  |  | 204.19 | 238.55 | 442.74 | 222.99 | 53.88 | 0.92 | 14.76 |  |
|  | B996-scht2 M1 | |  | 283.26 | 377.51 | 660.77 | 271.13 | 57.13 | 1.04 | 14.46 |  |
|  | B998-scht1-mand2 | |  | 227.47 | 286.83 | 514.30 | 216.13 | 55.77 | 1.05 | 15.96 |  |
|  | B998-scht2-mand1 | |  | 239.98 | 309.64 | 549.62 | 242.71 | 56.34 | 0.99 | 14.61 |  |
|  | MH-UdP LLM1 |  |  | 199.59 | 172.59 | 372.11 | 172.29 | 46.37 | 1.16 | 20.81 |  |
| Mean |  |  |  | 247.31 | 286.78 | 534.09 | 227.11 | 53.40 | 1.09 | 16.67 |  |
| SD |  |  |  | 40.01 | 63.51 | 100.82 | 26.07 | 3.00 | 0.12 | 2.07 |  |
| Range |  |  |  | 199.59-349.88 | 172.59-420.84 | 372.11-770.73 | 172.29-271.13 | 46.37-57.13 | 0.92-1.36 | 14.46-2081 |  |
| *H. antecessor* | AT6-5 | LM2 | 2 | 273.25 | 359.31 | 632.57 | 234.89 | 56.80 | 1.16 | 16.36 | Original data |
|  | AT6-113 |  | 2 | 259.81 | 288.47 | 548.28 | 207.70 | 52.61 | 1.25 | 18.93 |  |
|  | AT6-144 |  | 1 | 292.18 | 360.80 | 652.98 | 227.24 | 55.25 | 1.29 | 18.06 |  |
|  | ATD6-96 |  | 2 | 200.92 | 146.35 | 347.27 | 146.44 | 42.14 | 1.37 | 26.04 |  |
| Mean |  |  |  | 256.54 | 288.73 | 545.27 | 204.07 | 51.70 | 1.27 | 19.85 |  |
| SD |  |  |  | 34.11 | 87.25 | 120.87 | 34.72 | 5.72 | 0.07 | 3.69 |  |
| Range |  |  |  | 200.92-292.81 | 146.35-360.80 | 347.27-652.98 | 146.44-234.89 | 42.14-56.80 | 1.16-1.37 | 16.36-26.04 |  |
| NAH | Tighenif 2 |  |  | 373.06 | 502.42 | 875.48 | 312.54 | 57.39 | 1.19 | 15.01 | Zanolli and Mazurier [11] |
| HER | NG0802.3 |  |  | 240.80 | 219.20 | 460.00 | 181.10 | 47.65 | 1.33 | 22.05 | Zanolli [54] |
|  | NG92.3 |  |  | 266.60 | 257.20 | 523.80 | 199.20 | 49.10 | 1.34 | 21.04 |  |
|  | NG92D6ZE57s/d76 | |  | 279.70 | 291.90 | 571.60 | 222.20 | 51.07 | 1.26 | 18.98 |  |
|  | NG0802.2 |  |  | 260.10 | 219.50 | 479.60 | 182.70 | 45.77 | 1.42 | 23.60 |  |
| Mean |  |  |  | 261.80 | 246.95 | 508.75 | 196.30 | 48.40 | 1.34 | 21.42 |  |
| SD |  |  |  | 16.20 | 34.88 | 49.68 | 19.11 | 2.24 | 0.07 | 1.94 |  |
| Range |  |  |  | 240.8-279.7 | 219.2-291.9 | 460-571.6 | 181.1-222.2 | 45.76-51.06 | 1.25-1.42 | 18.97-23.60 |  |
| NEA | S36 |  |  | 169.13 | 266.97 | 436.10 | 189.75 | 61.22 | 0.89 | 13.84 | Olejniczak et al. [8] |
|  | KRD6 |  |  | 289.43 | 438.69 | 728.12 | 284.64 | 60.25 | 1.02 | 13.38 |  |
|  | KRD10 |  |  | 302.07 | 502.37 | 804.44 | 267.99 | 62.45 | 1.13 | 14.18 |  |
|  | KRP55 |  |  | 308.44 | 379.87 | 688.31 | 250.89 | 55.19 | 1.23 | 16.97 |  |
|  | KRP54 |  |  | 240.55 | 250.03 | 490.58 | 182.48 | 50.97 | 1.32 | 20.92 |  |
|  | KRD1 |  |  | 289.27 | 519.34 | 808.61 | 302.91 | 64.23 | 0.95 | 11.88 |  |
|  | 1 |  |  | 283.74 | 366.94 | 650.68 | 225.87 | 56.39 | 1.26 | 17.55 |  |
|  | 1 |  |  | 171.16 | 356.03 | 527.19 | 183.86 | 67.53 | 0.93 | 13.13 |  |
|  | 1 |  |  | 172.62 | 277.21 | 449.83 | 212.25 | 61.63 | 0.81 | 12.47 |  |
| Mean |  |  |  | 247.38 | 373.05 | 620.43 | 233.40 | 59.98 | 1.06 | 14.93 |  |
| SD |  |  |  | 60.35 | 98.98 | 147.91 | 45.30 | 5.03 | 0.18 | 2.95 |  |
| Range |  |  |  | 169.13-308.44 | 250.03-519.34 | 436.1-808.61 | 182.48-302.91 | 50.96-67.53 | 0.81-1.31 | 11.88-20.92 |  |
| MH | Bosco Pontini 1 | |  | 269.35 | 261.75 | 531.10 | 211.57 | 49.28 | 1.27 | 19.90 | Original data |
|  | Bosco Pontini 2 | |  | 236.36 | 229.80 | 466.16 | 197.28 | 49.30 | 1.20 | 19.56 |  |
|  | Bosco Pontini 4 | |  | 161.95 | 201.99 | 363.94 | 192.61 | 55.50 | 0.84 | 14.33 |  |
|  | B995 |  |  | 224.31 | 245.42 | 469.73 | 203.17 | 52.25 | 1.10 | 17.63 |  |
|  | San Canziano | |  | 257.58 | 241.99 | 499.57 | 208.55 | 48.44 | 1.24 | 19.82 |  |
|  | San Canziano | |  | 232.92 | 291.94 | 524.86 | 229.41 | 55.62 | 1.02 | 15.31 |  |
|  | Val Rosandra | |  | 239.71 | 266.34 | 506.06 | 226.53 | 52.63 | 1.06 | 16.45 |  |
|  |  |  |  | 235.72 | 244.00 | 479.72 | 191.25 | 50.86 | 1.23 | 19.72 | Weber and Bookstein [57] |
|  | MH-UdP |  |  | 201.13 | 180.94 | 382.07 | 181.02 | 47.36 | 1.11 | 19.64 | Original data |
|  | B996-24852 |  |  | 215.22 | 237.64 | 452.86 | 188.34 | 52.47 | 1.14 | 18.45 |  |
|  | B996-scht1 | |  | 342.90 | 426.01 | 768.91 | 285.35 | 55.40 | 1.20 | 15.97 |  |
|  | B996-scht1 | |  | 361.91 | 408.13 | 770.04 | 277.47 | 53.00 | 1.30 | 17.58 |  |
|  | B998-scht1-mand2 | |  | 267.22 | 267.12 | 534.34 | 195.45 | 49.99 | 1.37 | 21.23 |  |
|  | UCL9 M2d |  |  | 276.83 | 283.21 | 560.04 | 234.66 | 50.57 | 1.18 | 17.96 |  |
|  | UCL9 M2g |  |  | 293.27 | 323.23 | 616.50 | 254.21 | 52.43 | 1.15 | 16.81 |  |
|  | MH-UTP |  |  | 228.47 | 190.32 | 418.80 | 171.19 | 45.45 | 1.33 | 23.20 |  |
|  | MH-MNHN | |  | 300.37 | 326.87 | 627.24 | 236.46 | 52.11 | 1.27 | 18.44 |  |
|  |  |  |  | 181.94 | 135.34 | 317.28 | 282.04 | 42.66 | 0.65 | 12.56 | Olejniczak et al. [8] |
|  |  |  |  | 219.62 | 180.82 | 400.44 | 95.40 | 45.16 | 2.30 | 40.71 |  |
|  |  |  |  | 191.51 | 240.61 | 432.12 | 128.45 | 55.68 | 1.49 | 23.97 |  |
|  |  |  |  | 227.43 | 236.21 | 463.64 | 119.93 | 50.95 | 1.90 | 30.68 |  |
|  |  |  |  | 221.31 | 249.40 | 470.71 | 196.79 | 52.98 | 1.12 | 17.87 |  |
|  |  |  |  | 232.00 | 259.20 | 491.20 | 216.79 | 52.77 | 1.07 | 16.78 |  |
|  |  |  |  | 239.77 | 263.90 | 503.67 | 180.33 | 52.40 | 1.33 | 20.73 |  |
|  |  |  |  | 252.98 | 339.76 | 592.74 | 141.70 | 57.32 | 1.79 | 25.59 |  |
|  |  |  |  | 354.43 | 305.28 | 659.71 | 238.64 | 46.27 | 1.49 | 22.06 |  |
| Mean |  |  |  | 248.70 | 262.97 | 511.67 | 203.25 | 51.11 | 1.27 | 20.11 |  |
| SD |  |  |  | 49.75 | 65.53 | 110.61 | 47.55 | 3.63 | 0.33 | 5.62 |  |
| Range |  |  |  | 161.94-361.91 | 135.4-426.01 | 317.28-770.04 | 95.4-285.35 | 42.65-57.32 | 0.65-2.3 | 12.56-40.71 |  |
| *H. antecessor* | AT6-5 | LM3 | 1 | 229.21 | 261.93 | 491.15 | 191.97 | 53.33 | 1.19 | 18.66 | Original data |
|  | ATD6-96 |  | 2 | 109.39 | 55.52 | 164.91 | 81.15 | 33.67 | 1.35 | 35.33 |  |
|  | AT6-113 |  | 1 | 210.24 | 185.64 | 395.88 | 158.77 | 46.89 | 1.32 | 23.21 |  |
| Mean |  |  |  | 182.95 | 167.70 | 350.65 | 143.96 | 44.63 | 1.29 | 25.74 |  |
| SD |  |  |  | 64.41 | 104.37 | 167.76 | 56.88 | 10.02 | 0.08 | 8.62 |  |
| Range |  |  |  | 109.39-229.21 | 55.52-261.93 | 164.91-491.15 | 81.15-191.97 | 33.67-53.33 | 1.19-1.35 | 18.66-35.33 |  |
| NAH | Tighenif 2 |  |  | 372.24 | 371.53 | 743.77 | 255.48 | 49.95 | 1.46 | 20.27 | Zanolli and Mazurier [11] |
| NEA | BD01 |  |  | 154.04 | 270.31 | 424.35 | 187.03 | 63.70 | 0.82 | 12.74 | Olejniczak et al. [8] |
|  | S36 |  |  | 163.18 | 244.62 | 407.80 | 177.94 | 59.99 | 0.92 | 14.66 |  |
|  | S43 |  |  | 185.04 | 285.54 | 470.58 | 142.85 | 60.68 | 1.30 | 19.67 |  |
|  | KRD9 |  |  | 204.74 | 343.83 | 548.57 | 228.03 | 62.68 | 0.90 | 12.82 |  |
|  | KRP57 |  |  | 246.77 | 259.29 | 506.06 | 192.94 | 51.24 | 1.28 | 20.06 |  |
|  | KRPD85 |  |  | 262.18 | 253.05 | 515.23 | 186.03 | 49.11 | 1.41 | 22.28 |  |
|  | Q760-H9 |  |  | 222.11 | 485.90 | 708.01 | 192.52 | 68.63 | 1.15 | 14.67 |  |
|  | 1 |  |  | 194.84 | 271.89 | 466.73 | 181.20 | 58.25 | 1.08 | 16.60 |  |
|  | CG XII |  |  | 239.40 | 300.14 | 539.54 | 206.76 | 55.63 | 1.16 | 17.29 |  |
| Mean |  |  |  | 208.03 | 301.62 | 509.65 | 188.37 | 58.88 | 1.11 | 16.75 |  |
| SD |  |  |  | 37.51 | 75.22 | 88.61 | 22.88 | 6.14 | 0.20 | 3.37 |  |
| Range |  |  |  | 154.04-262.18 | 244.62-485.90 | 407.80-708.01 | 142.85-228.03 | 49.11-68.62 | 0.82-1.40 | 12.73-22.28 |  |
|  |  |  |  |  |  |  |  |  |  |  |  |
| MH | B998-scht3 |  |  | 245.64 | 228.53 | 474.17 | 192.40 | 48.20 | 1.28 | 20.88 | Original data |
|  | B996-scht1 | |  | 343.86 | 328.87 | 672.73 | 243.27 | 48.89 | 1.41 | 20.48 |  |
|  | San Canziano | |  | 226.14 | 190.69 | 416.83 | 185.30 | 45.75 | 1.22 | 21.20 |  |
|  | MH-CZ |  |  | 243.12 | 237.26 | 480.37 | 191.65 | 49.39 | 1.27 | 20.49 |  |
|  | MH-UdP |  |  | 206.51 | 170.48 | 376.99 | 178.26 | 45.22 | 1.16 | 20.89 |  |
|  | B996-scht1 | |  | 362.14 | 405.48 | 767.63 | 273.48 | 52.82 | 1.32 | 17.89 |  |
|  | B996-scht2 | |  | 254.55 | 288.75 | 543.30 | 216.63 | 53.15 | 1.18 | 17.78 |  |
|  | B998-scht2-mand2 | |  | 166.58 | 167.74 | 334.32 | 153.79 | 50.17 | 1.08 | 19.64 |  |
|  |  |  |  | 178.53 | 149.70 | 328.23 | 121.09 | 45.61 | 1.47 | 27.77 | Olejniczak et al. [8] |
|  |  |  |  | 181.56 | 146.81 | 328.37 | 125.05 | 44.71 | 1.45 | 27.52 |  |
|  |  |  |  | 196.10 | 242.06 | 438.16 | 149.94 | 55.24 | 1.31 | 20.99 |  |
|  |  |  |  | 263.03 | 250.84 | 513.87 | 182.51 | 48.81 | 1.44 | 22.85 |  |
|  |  |  |  | 299.26 | 372.37 | 671.63 | 162.05 | 55.44 | 1.85 | 25.67 |  |
| Mean |  |  |  | 243.62 | 244.58 | 488.20 | 182.72 | 49.49 | 1.34 | 21.85 |  |
| SD |  |  |  | 61.68 | 83.91 | 142.74 | 43.51 | 3.72 | 0.19 | 3.25 |  |
| Range |  |  |  | 166.58-362.14 | 146.81-405.48 | 328.23-767.63 | 121.09-273.48 | 44.70-55.44 | 1.08-1.85 | 17.78-27.77 |  |

Upper molars: *H. antecessor* from Gran Dolina (original data). HER: *H. erectus* (Sangiran_M1, Zanolli [54]). NEA: Neanderthals (Olejniczak et al.[8]; Bayle et al. [55]. MH: modern humans (Olejniczak et al. [8]). Lower molars: *H. antecessor* from Gran Dolina (original data). EAH: East African *Homo* (Eritrea_M1, Zanolli et al. [56]). NAH: North African *Homo* (Tighenif_M2&M3, Zanolli and Mazurier [11]). HER: *H. erectus* (Sangiran, Zanolli [54]). NEA: Neanderthals (Olejniczak et al. [8]). MH: modern humans (Olejniczak et al. [8]; Weber and Bookstein [57] and original data).
