# Supplementary material for: Tooth crown tissue proportions and enamel thickness in Early Pleistocene Homo antecessor molars (Atapuerca, Spain)
Source: PLoS One. 2018 Oct 3;13(10):e0203334. doi: 10.1371/journal.pone.0203334 (PMC6169863; doi:10.1371/journal.pone.0203334)
Supplement: S3 Table — Upper molars: H. antecessor from Gran Dolina (original data). NAH: North African Homo (Tighenif_M2&M3, Zanolli and Mazurier [11]). EMPH: European Middle Pleistocene Homo (Visogliano6_M1 & Visogliano3_M2, Zanolli et al. [17]). NEA: Neanderthals (original data). MH: modern humans (European origin, original data). Lower molars: H. antecessor from Gran Dolina (original data). NAH: North African Homo (Tighenif_M1&M3; Zanolli and Mazurier [11]). HER: H. erectus (Sangiran_M1&M2; HER: H. erectus (Sangiran, Zanolli [54]). EMPH: European Middle Pleistocene Homo (Fontana Ranuccio_M1, Zanolli et al. [17]).NEA: Neanderthals (original data). MH: modern humans (European origin, original data and Weber and Bookstein [57]). (DOCX) [file pone.0203334.s004.docx]

S3 Table. 3D lateral enamel thickness values measured in the TD6 maxillary and mandibular molars and those of the extinct and extant specimens/populations

| **Taxon/group** |  | **Tooth** | **Wear** | **LVe (mm3)** | **LVcdp (mm3)** | **LVc (mm3)** | **LSEDJ (mm2)** | **LVcdp/Vc (%)** | **L3D AET (mm)** | **L3D RET** | **Data Source** |
| --- | --- | --- | --- | --- | --- | --- | --- | --- | --- | --- | --- |
| *H. antecessor* | ATD6-10 | UM1 | 3 | 79.59 | 270.66 | 350.25 | 110.49 | 77.28 | 0.72 | 11.14 | Original Data |
|  | ATD6-11 |  | 3 | 83.46 | 305.42 | 388.88 | 123.32 | 78.54 | 0.68 | 10.05 |  |
|  | ATD6-69 |  | 2 | 71.81 | 235.23 | 307.04 | 99.59 | 76.61 | 0.72 | 11.68 |  |
|  | ATD6-103 |  | 2 | 71.91 | 324.14 | 396.05 | 126.00 | 81.84 | 0.57 | 8.31 |  |
| Mean |  |  |  | 76.69 | 283.86 | 360.56 | 114.85 | 78.57 | 0.67 | 10.29 |  |
| SD |  |  |  | 5.80 | 39.27 | 40.96 | 12.22 | 2.33 | 0.07 | 1.49 |  |
| Range |  |  |  | 71.81-83.46 | 235.23-324.14 | 307.04-396.05 | 99.59-126.00 | 76.61-81.84 | 0.57-0.72 | 8.31-11.68 |  |
| European *Homo* | Visogliano 6 |  |  | 86.20 | 264.45 | 350.65 | 121.41 | 75.42 | 0.71 | 11.06 | Zanolli et al. [17] |
| African *Homo* | Tighenif |  |  | 116.99 | 398.79 | 515.78 | 172.25 | 77.32 | 0.68 | 9.23 | Zanolli and Mazurier [11] |
| Neanderthal | KRD101 |  |  | 51.29 | 210.21 | 261.50 | 96.00 | 80.39 | 0.53 | 8.99 | Original Data |
|  | D134 |  |  | 97.40 | 352.44 | 449.85 | 140.90 | 78.35 | 0.69 | 9.79 |  |
|  | D136 |  |  | 112.46 | 414.76 | 527.22 | 167.39 | 78.67 | 0.67 | 9.01 |  |
|  | D171 |  |  | 96.30 | 287.46 | 383.76 | 133.49 | 74.91 | 0.72 | 10.93 |  |
|  | D174 |  |  | 87.54 | 260.07 | 347.61 | 126.63 | 74.82 | 0.69 | 10.83 |  |
|  | D16 |  |  | 81.13 | 285.00 | 366.13 | 128.06 | 77.84 | 0.63 | 9.63 |  |
| Mean |  |  |  | 87.69 | 301.66 | 389.35 | 132.08 | 77.49 | 0.66 | 9.86 |  |
| SD |  |  |  | 20.74 | 72.03 | 90.92 | 23.13 | 2.21 | 0.07 | 0.85 |  |
| Range |  |  |  | 51.29-112.46 | 210.21-414.76 | 261.50-527.22 | 96.00-167.39 | 74.82-80.39 | 0.53-0.72 | 8.99-10.93 |  |
| *H. sapiens* | Bosco Pontini 9 | |  | 72.23 | 214.05 | 286.28 | 117.84 | 74.77 | 0.61 | 10.25 | Original Data |
|  | Bosco Pontini 8 | |  | 70.24 | 213.00 | 283.24 | 115.22 | 75.20 | 0.61 | 10.21 |  |
|  | Pigorini |  |  | 74.20 | 225.37 | 299.57 | 118.59 | 75.23 | 0.63 | 10.28 |  |
|  | Pigorini |  |  | 61.28 | 218.09 | 279.38 | 114.22 | 78.06 | 0.54 | 8.91 |  |
|  | Bosco Pontini 6 | |  | 56.40 | 191.72 | 248.12 | 104.52 | 77.27 | 0.54 | 9.36 |  |
|  | Bosco Pontini 6 | |  | 61.59 | 186.41 | 248.00 | 110.84 | 75.17 | 0.56 | 9.73 |  |
| Mean |  |  |  | 65.99 | 208.11 | 274.10 | 113.54 | 75.95 | 0.58 | 9.79 |  |
| SD |  |  |  | 7.18 | 15.47 | 21.28 | 5.21 | 1.36 | 0.04 | 0.56 |  |
| Range |  |  |  | 56.40-74.20 | 186.41-225.37 | 248.00-299.57 | 104.52-118.59 | 74.77-78.06 | 0.54-0.63 | 8.91-10.28 |  |
| *H. antecessor* | ATD6-12 | UM2 | 2 | 66.08 | 274.03 | 340.11 | 105.42 | 80.57 | 0.63 | 9.65 | Original Data |
| *H. antecessor* | ATD6-69 |  | 1 | 61.77 | 224.72 | 286.49 | 84.60 | 78.44 | 0.73 | 12.01 |  |
| Mean |  |  |  | 63.93 | 249.38 | 313.30 | 95.01 | 79.51 | 0.68 | 10.83 |  |
| SD |  |  |  | 3.05 | 34.87 | 37.92 | 14.72 | 1.51 | 0.07 | 1.67 |  |
| European *Homo* | Visogliano3 |  |  | 76.34 | 242.16 | 318.50 | 134.09 | 76.03 | 0.57 | 9.13 | Zanolli et al. [17] |
| Neanderthal | KRD98 |  |  | 56.04 | 199.03 | 255.07 | 100.46 | 78.03 | 0.56 | 9.55 | Original Data |
|  | D96 |  |  | 64.04 | 242.31 | 306.35 | 107.22 | 79.09 | 0.60 | 9.58 |  |
|  | D135 |  |  | 63.55 | 678.03 | 741.58 | 109.09 | 91.43 | 0.58 | 6.63 |  |
|  | D165 |  |  | 73.34 | 246.52 | 319.85 | 114.40 | 77.07 | 0.64 | 10.22 |  |
|  | D166 |  |  | 94.32 | 363.56 | 457.89 | 140.79 | 79.40 | 0.67 | 9.39 |  |
|  | D169 |  |  | 86.00 | 252.63 | 338.63 | 123.33 | 74.60 | 0.70 | 11.03 |  |
| Mean |  |  |  | 72.88 | 330.35 | 403.23 | 115.88 | 79.94 | 0.62 | 9.40 |  |
| SD |  |  |  | 14.70 | 178.91 | 178.84 | 14.40 | 5.89 | 0.05 | 1.49 |  |
| Range |  |  |  | 56.04-94.32 | 199.03-678.03 | 255.07-741.58 | 100.46-140.79 | 74.60-91.43 | 0.56-0.70 | 6.63-11.03 |  |
| *H. sapiens* | Bosco Pontini 5 | |  | 35.15 | 130.38 | 165.53 | 73.01 | 78.77 | 0.48 | 9.49 | Original Data |
|  | Bosco Pontini 6 | |  | 24.50 | 95.62 | 120.12 | 60.46 | 79.60 | 0.41 | 8.86 |  |
|  | Bosco Pontini 9 | |  | 53.52 | 171.71 | 225.23 | 92.65 | 76.24 | 0.58 | 10.39 |  |
|  | Pigorini |  |  | 29.97 | 127.78 | 157.76 | 66.45 | 81.00 | 0.45 | 8.95 |  |
|  | Pigorini |  |  | 33.78 | 137.08 | 170.86 | 68.72 | 80.23 | 0.49 | 9.53 |  |
| Mean |  |  |  | 92.76 | 132.52 | 167.90 | 72.26 | 79.17 | 0.48 | 9.45 |  |
| SD |  |  |  | 35.35 | 27.14 | 37.71 | 12.27 | 1.83 | 0.06 | 0.61 |  |
| Range |  |  |  | 24.50-53.52 | 95.62-171.71 | 120.12-225.23 | 60.46-92.65 | 76.24-81.00 | 0.41-0.58 | 8.86-10.39 |  |
| **Taxon/group** |  | **Tooth** | **Wear** | **LVe (mm3)** | **LVcdp (mm3)** | **LVc (mm3)** | **LSEDJ (mm2)** | **LVcdp/Vc (%)** | **L3D AET (mm)** | **L3D RET** | **Data Source** |
| *H. antecessor* | AT6-5 | LM1 | 3 | 59.71 | 250.86 | 310.57 | 100.01 | 80.77 | 0.60 | 9.47 | Original Data |
|  | AT6-94 |  | 2 | 64.47 | 333.63 | 398.10 | 125.71 | 83.81 | 0.51 | 7.39 |  |
|  | AT6-112 |  | 1 | 56.72 | 263.13 | 319.85 | 106.11 | 82.27 | 0.53 | 8.34 |  |
|  | ATD6-96 |  | 3 | 37.48 | 144.82 | 182.30 | 58.12 | 79.44 | 0.64 | 12.28 |  |
| Mean |  |  |  | 54.60 | 248.11 | 302.70 | 97.49 | 81.57 | 0.57 | 9.37 |  |
| SD |  |  |  | 11.85 | 77.92 | 89.36 | 28.44 | 1.88 | 0.06 | 2.12 |  |
| Range |  |  |  | 37.48-64.47 | 144.82-333.627 | 182.30-398.10 | 58.12-125.71 | 79.44-82.27 | 0.51-0.64 | 7.39-12.28 |  |
| NAH | Tighenif 2 |  |  | 102.82 | 389.11 | 491.93 | 151.14 | 79.10 | 0.68 | 9.32 | Original Data |
| HER | NG92.2 |  |  | 44.20 | 186.33 | 230.53 | 85.56 | 80.83 | 0.52 | 9.05 | Original Data |
| EMPH | FR1R |  |  | 60.77 | 239.61 | 300.37 | 110.08 | 79.77 | 0.55 | 8.89 | Original Data |
| NEA | D81 |  |  | 68.28 | 294.34 | 362.62 | 134.28 | 81.17 | 0.51 | 7.64 | Original Data |
|  | D77 |  |  | 94.41 | 363.66 | 458.07 | 152.79 | 79.39 | 0.62 | 8.66 |  |
|  | D79 |  |  | 104.03 | 404.52 | 508.55 | 159.88 | 79.54 | 0.65 | 8.80 |  |
|  | D80 |  |  | 108.44 | 306.13 | 414.57 | 138.06 | 73.84 | 0.79 | 11.65 |  |
|  | D105 |  |  | 98.69 | 379.77 | 478.46 | 149.37 | 79.37 | 0.66 | 9.12 |  |
|  | BDJ4C9 |  |  | 58.26 | 247.55 | 305.81 | 114.03 | 80.95 | 0.51 | 8.14 |  |
|  | S5 |  |  | 62.24 | 246.12 | 308.37 | 115.39 | 79.82 | 0.54 | 8.61 |  |
|  | S14-7 |  |  | 56.11 | 239.88 | 295.98 | 98.95 | 81.04 | 0.57 | 9.13 |  |
|  | S49 |  |  | 58.00 | 252.98 | 310.97 | 110.01 | 81.35 | 0.53 | 8.34 |  |
|  | Regourdou 1 |  |  | 36.07 | 205.37 | 241.45 | 92.80 | 85.06 | 0.39 | 6.59 |  |
| Mean |  |  |  | 74.45 | 294.03 | 368.49 | 126.56 | 80.15 | 0.58 | 8.67 |  |
| SD |  |  |  | 24.82 | 67.89 | 90.76 | 23.49 | 2.78 | 0.11 | 1.30 |  |
| Range |  |  |  | 36.07-108.44 | 205.37-404.52 | 241.45-508.55 | 92.80-159.88 | 73.84-85.06 | 0.39-0.79 | 6.59-11.65 |  |
| MH | Bosco Pontini 1 | |  | 53.97 | 197.08 | 251.05 | 99.21 | 78.50 | 0.54 | 9.35 | Original Data |
|  | Bosco Pontini 2 | |  | 63.73 | 212.31 | 276.04 | 103.55 | 76.91 | 0.62 | 10.32 |  |
|  | B996_scht1 |  |  | 113.81 | 362.55 | 476.37 | 156.12 | 76.11 | 0.73 | 10.22 |  |
|  | B996_scht2 |  |  | 76.49 | 285.87 | 362.36 | 123.97 | 78.89 | 0.62 | 9.37 |  |
|  | B998_scht1_Mand2 | |  | 63.86 | 210.78 | 274.64 | 104.17 | 76.75 | 0.61 | 10.30 |  |
|  | B998-scht2-mand1 | |  | 49.26 | 228.13 | 277.39 | 101.01 | 82.24 | 0.49 | 7.98 |  |
|  | B998-scht2-mand3 | |  | 43.12 | 205.51 | 248.63 | 96.85 | 82.66 | 0.45 | 7.54 |  |
|  | San Canziano | |  | 51.38 | 203.73 | 255.11 | 93.23 | 79.86 | 0.55 | 9.37 |  |
|  | U21 |  |  | 48.65 | 168.99 | 217.64 | 80.27 | 50.00 | 0.61 | 10.96 |  |
|  | U57 |  |  | 14.91 | 52.61 | 67.51 | 38.17 | 50.00 | 0.39 | 10.42 |  |
|  | UTP |  |  | 46.20 | 179.27 | 225.47 | 87.80 | 50.00 | 0.53 | 9.33 |  |
|  | Sbg2 |  |  | 34.45 | 123.60 | 158.04 | 67.85 | 50.00 | 0.51 | 10.19 |  |
|  | Sbg4 |  |  | 51.38 | 160.98 | 212.35 | 84.59 | 50.00 | 0.61 | 11.17 |  |
| Mean |  |  |  | 54.71 | 161.66 | 216.37 | 95.14 | 67.84 | 0.56 | 11.81 |  |
| SD |  |  |  | 23.14 | 110.34 | 129.85 | 27.59 | 14.80 | 0.09 | 3.42 |  |
| Range |  |  |  | 14.91-113.81 | 52.61-362.55 | 67.51-476.37 | 38.17-156.12 | 50.00-82.66 | 0.39-0.79 | 7.54-11.81 |  |
| *H. antecessor* | AT6-5 | LM2 | 3 | 55.69 | 297.87 | 353.56 | 103.67 | 84.25 | 0.54 | 8.04 | Original Data |
|  | ATD6-96 |  | 2 | 22.26 | 88.12 | 110.38 | 35.98 | 79.83 | 0.62 | 13.90 |  |
|  | ATD6-144 |  | 2 | 58.74 | 270.53 | 329.27 | 107.78 | 82.16 | 0.54 | 8.43 |  |
|  | ATD6-113 |  | 2 | 48.47 | 219.90 | 268.37 | 83.65 | 81.94 | 0.58 | 9.60 |  |
| Mean |  |  |  | 46.29 | 219.11 | 265.40 | 82.77 | 82.05 | 0.57 | 9.99 |  |
| SD |  |  |  | 16.59 | 93.11 | 109.38 | 32.93 | 1.80 | 0.04 | 2.69 |  |
| Range |  |  |  | 22.26-58.74 | 88.12-297.83 | 110.38-353.56 | 35.98-107.78 | 79.83-84.25 | 0.54-0.62 | 8.04-13.90 |  |
| HER | NG92.3 |  |  | 51.89 | 244.38 | 296.27 | 93.57 | 82.48 | 0.55 | 8.87 | Original Data |
|  | NG92 D6 ZE 57 s/d 76 | |  | 50.75 | 216.19 | 266.95 | 99.70 | 80.99 | 0.51 | 8.48 |  |
|  | NG0802.2 |  |  | 42.66 | 170.73 | 213.39 | 78.85 | 80.01 | 0.54 | 9.75 |  |
| Mean |  |  |  | 48.43 | 210.43 | 258.87 | 90.71 | 81.16 | 0.53 | 9.03 |  |
| SD |  |  |  | 5.03 | 37.16 | 42.03 | 10.72 | 1.25 | 0.02 | 0.65 |  |
| Min |  |  |  | 42.66 | 170.73 | 213.39 | 78.85 | 80.01 | 0.51 | 8.48 |  |
| Max |  |  |  | 51.89 | 244.38 | 296.27 | 99.70 | 82.48 | 0.55 | 9.75 |  |
| Range |  |  |  | 42.66-51.89 | 170.73-244.38 | 213.39-296.27 | 78.85-99.70 | 80.01-82.48 | 0.51-0.55 | 8.48-9.75 |  |
| NEA | D6 |  |  | 63.77 | 344.51 | 408.28 | 147.03 | 84.38 | 0.43 | 6.19 | Original Data |
|  | D86 |  |  | 84.59 | 346.71 | 431.30 | 135.22 | 80.39 | 0.63 | 8.91 |  |
|  | D107 |  |  | 110.62 | 422.03 | 532.65 | 145.90 | 79.23 | 0.76 | 10.11 |  |
|  | D10 |  |  | 106.00 | 358.00 | 464.00 | 156.14 | 77.15 | 0.68 | 9.56 |  |
|  | D1 |  |  | 83.93 | 380.47 | 464.39 | 138.97 | 81.93 | 0.60 | 8.33 |  |
|  | Regourdou |  |  | 49.51 | 241.17 | 290.68 | 97.96 | 82.97 | 0.51 | 8.12 |  |
|  | Regourdou |  |  | 47.52 | 230.45 | 277.97 | 93.32 | 82.91 | 0.51 | 8.31 |  |
| Mean |  |  |  | 77.99 | 331.90 | 409.90 | 130.65 | 81.28 | 0.59 | 8.50 |  |
| SD |  |  |  | 25.40 | 70.79 | 94.03 | 24.84 | 2.50 | 0.11 | 1.25 |  |
| Range |  |  |  | 47.52-110.62 | 230.45-422.03 | 277.97-532.65 | 93.32-156.14 | 77.15-84.38 | 0.43-0.76 | 6.19-10.11 |  |
| MH | B995 LRM2 |  |  | 34.39 | 163.23 | 197.62 | 74.47 | 82.60 | 0.46 | 8.45 | Original Data |
|  | B996 24852 |  |  | 46.83 | 185.98 | 232.81 | 91.96 | 79.89 | 0.51 | 8.92 |  |
|  | B996-scht1 |  |  | 90.83 | 351.01 | 441.84 | 136.08 | 79.44 | 0.67 | 9.46 |  |
|  | B996-scht1 |  |  | 87.99 | 318.78 | 406.77 | 128.32 | 78.37 | 0.69 | 10.04 |  |
|  | B998-scht1-mand2 | |  | 61.65 | 177.43 | 239.08 | 82.79 | 74.21 | 0.74 | 13.25 |  |
|  | MH-MNHN |  |  | 50.48 | 225.67 | 276.15 | 89.42 | 81.72 | 0.56 | 9.27 |  |
|  | LM2 |  |  | 54.53 | 199.05 | 253.58 | 85.50 | 78.50 | 0.64 | 10.92 | Weber and Bookstein [57] |
|  | Bosco Pontini 1 | |  | 43.37 | 158.37 | 201.75 | 74.54 | 78.50 | 0.58 | 10.75 | Original Data |
|  | Bosco Pontini 2 | |  | 45.06 | 155.67 | 200.73 | 76.91 | 77.55 | 0.59 | 10.89 |  |
|  | Bosco Pontini 4 | |  | 32.14 | 123.44 | 155.57 | 64.89 | 79.34 | 0.50 | 9.95 |  |
| Mean |  |  |  | 54.73 | 205.86 | 260.59 | 90.49 | 79.01 | 0.59 | 10.19 |  |
| SD |  |  |  | 20.23 | 73.67 | 92.97 | 23.45 | 2.29 | 0.09 | 1.37 |  |
| Range |  |  |  | 32.14-90.83 | 123.44-351.01 | 155.57-441.84 | 64.89-136.08 | 74.21-82.60 | 0.46-0.74 | 8.45-13.25 |  |
| *H. antecessor* | ATD6-5 | LM3 |  | 39.16 | 212.36 | 251.52 | 80.55 | 84.43 | 0.49 | 8.15 | Original Data |
|  | ATD6-96 |  |  | 6.91 | 26.48 | 33.39 | 17.21 | 79.31 | 0.40 | 13.47 |  |
|  | ATD6-113 |  |  | 25.34 | 127.31 | 152.65 | 51.24 | 83.40 | 0.49 | 9.83 |  |
| Mean |  |  |  | 23.80 | 122.05 | 145.85 | 49.67 | 82.38 | 0.46 | 10.48 |  |
| SD |  |  |  | 16.18 | 93.05 | 109.22 | 31.70 | 2.71 | 0.05 | 2.72 |  |
| Range |  |  |  | 6.91-39.16 | 26.48-212.36 | 33.39-251.52 | 17.21-80.55 | 17.21-80.55 | 0.40-0.49 | 8.15-13.47 |  |
| HER | NG9107.2 |  |  | 29.41 | 145.13 | 174.54 | 64.21 | 83.15 | 0.46 | 8.72 | Original Data |
|  | Tighenif 2 |  |  | 79.89 | 302.65 | 382.54 | 124.33 | 79.12 | 0.64 | 9.57 |  |
| NEA | KRD5 |  |  | 67.70 | 276.21 | 343.92 | 123.28 | 80.31 | 0.55 | 8.43 |  |
|  | KRD7 |  |  | 83.44 | 232.45 | 315.89 | 116.58 | 73.59 | 0.72 | 11.64 | Original Data |
|  | KRD85 |  |  | 66.24 | 195.19 | 261.43 | 98.02 | 74.66 | 0.68 | 11.65 |  |
|  | KRD106 |  |  | 80.33 | 314.03 | 394.36 | 132.28 | 79.63 | 0.61 | 8.93 |  |
|  | Regourdou |  |  | 51.51 | 219.06 | 270.57 | 98.97 | 80.96 | 0.52 | 8.63 |  |
|  | Regourdou |  |  | 48.02 | 211.39 | 259.42 | 97.61 | 81.49 | 0.49 | 8.26 |  |
| Mean |  |  |  | 66.21 | 241.39 | 307.60 | 111.12 | 78.44 | 0.59 | 9.59 |  |
| SD |  |  |  | 14.46 | 44.95 | 54.29 | 15.01 | 3.42 | 0.09 | 1.61 |  |
| Range |  |  |  | 29.41-83.44 | 145.13-314.03 | 174.54-394.36 | 64.21-132.28 | 73.59-83.15 | 0.46-0.72 | 8.26-11.65 |  |
| MH | MH-UdP |  |  | 38.24 | 135.23 | 173.46 | 81.94 | 77.96 | 0.47 | 9.09 | Original Data |
|  | MH-CZ |  |  | 49.39 | 228.74 | 278.13 | 99.06 | 82.24 | 0.50 | 8.15 |  |
|  | B996 scht1 |  |  | 79.90 | 314.86 | 394.75 | 131.75 | 79.76 | 0.61 | 8.91 |  |
|  | B998 scht2 |  |  | 51.00 | 191.97 | 242.98 | 85.40 | 79.01 | 0.60 | 10.35 |  |
|  | B998-scht2-mand2 | |  | 22.25 | 130.04 | 152.29 | 69.99 | 85.39 | 0.32 | 6.28 |  |
|  | B996-scht1 |  |  | 75.47 | 262.70 | 338.16 | 116.24 | 77.68 | 0.65 | 10.14 |  |
| Mean |  |  |  | 52.71 | 210.59 | 263.30 | 97.40 | 80.34 | 0.52 | 8.82 |  |
| SD |  |  |  | 21.95 | 72.71 | 93.71 | 23.12 | 2.96 | 0.12 | 1.49 |  |
| Range |  |  |  | 22.25-79.90 | 130.04-314.86 | 152.29-394.75 | 69.99-131.75 | 77.68-85.39 | 0.32-0.65 | 6.28-10.35 |  |

Upper molars: *H. antecessor* from Gran Dolina (original data). NAH: North African *Homo* (Tighenif_M2&M3, Zanolli and Mazurier [11]). EMPH: European Middle Pleistocene *Homo* (Visogliano6_M1 & Visogliano3_M2, Zanolli et al. [17]). NEA: Neanderthals (original data). MH: modern humans (European origin, original data). Lower molars: *H. antecessor* from Gran Dolina (original data). NAH: North African *Homo* (Tighenif_M1&M3; Zanolli and Mazurier [11]). HER: *H. erectus* (Sangiran_M1&M2; HER: *H. erectus* (Sangiran, Zanolli [54]). EMPH: European Middle Pleistocene *Homo* (Fontana Ranuccio_M1, Zanolli et al. [17]).NEA: Neanderthals (original data). MH: modern humans (European origin, original data and Weber and Bookstein [57]).
